# The Novel HSF1 Inhibitor NXP800 Exhibits Robust Antitumor Activity in Hepatocellular Carcinoma

**DOI:** 10.3390/ijms27062781

**Published:** 2026-03-19

**Authors:** Sara M. Steinmann, Melania Lazzari, Augustinus Kleinle, Dora Pischedda, Antonio Cigliano, Grazia Galleri, Heiko Siegmund, Claudia Fischer, Salvatore Piscuoglio, Matthias Evert, Diego F. Calvisi

**Affiliations:** 1 Institute of Pathology, University of Regensburg, Franz-Josef-Strauß-Allee 11, 93053 Regensburg, Germany; sara.steinmann@ur.de (S.M.S.); augustinus.kleinle@stud.uni-regensburg.de (A.K.); heiko.sigmund@ur.de (H.S.); claudia.fisher@ur.de (C.F.); matthias.evert@ur.de (M.E.); 2 Department of Internal Medicine I, Gastroenterology, Hepatology, Endocrinology, Rheumatology, and Infectious Diseases, University Hospital Regensburg, Franz-Josef-Strauß-Allee 11, 93053 Regensburg, Germany; 3 Department of Biomedical Science, Humanitas University, Via Rita Levi Montalcini 4, 20072 Pieve Emanuele, Italy; melania.lazzari@humanitasresearch.it (M.L.); salvatore.piscuoglio@hunimed.eu (S.P.); 4 IRCCS Humanitas Research Hospital, Via Manzoni 56, 20089 Rozzano, Italy; 5 Visceral Surgery and Precision Medicine Research Laboratory, Department of Biomedicine, University of Basel, 4001 Basel, Switzerland; 6 Department of Medicine, Surgery, and Pharmacy, University of Sassari, 07100 Sassari, Italy; d.pischedda@studenti.uniss.it (D.P.); antonio.cigliano@unicamillus.org (A.C.); 7 Faculty of Medicine, Saint Camillus International University of Health Sciences, 00131 Rome, Italy; 8 Department of Biomedical Sciences, University of Sassari, 07100 Sassari, Italy; galleri@uniss.it

**Keywords:** HSF1, NXP800, CCT361814, HSF1 pathway inhibitor, heat shock response, hepatocellular carcinoma, HCC

## Abstract

Heat-shock factor 1 (HSF1) is a multifunctional transcription factor whose overexpression is associated with the development, progression, and aggressiveness of several tumor types, including hepatocellular carcinoma (HCC). In the present study, we thoroughly investigated the antitumor activity of NXP800, a recently developed HSF1 inhibitor that is currently tested in clinical trials, on HCC growth. We discovered that NXP800 inhibits the cell growth of human HCC cell lines by reducing proliferation, inducing apoptosis, and causing DNA damage. At the metabolic level, NXP800 significantly decreased mitochondrial respiration, which was associated with extensive structural alterations in the mitochondria, and reduced glycolysis of HCC cells. At the molecular level, NXP800 administration led to the upregulation of the integrated stress response and downregulation of the E2F1 signaling cascade. In addition, NXP800 profoundly constrained the growth of HCC patient-derived organoids. Furthermore, NXP800 antitumor properties were significantly augmented when NXP800 was coupled with the DNA-damaging agent doxorubicin or the PARP inhibitor olaparib. Our investigation indicates that NXP800 has significant antitumor activity and might represent a promising therapeutic agent for the treatment of human HCC.

## 1. Introduction

Hepatocellular carcinoma (HCC) is the most common type of primary liver cancer, accounting for approximately 85% of cases. It ranks as the sixth most common cancer and is the third leading cause of cancer-related deaths worldwide, making it a significant global health concern [1]. Several potentially curative treatments are available for early-stage HCC, including surgical resection, radiofrequency ablation, transarterial chemoembolization, and liver transplantation [2,3]. However, due to the lack of specific symptoms, many patients are diagnosed at advanced stages of the disease, when the tumor cannot be successfully eradicated. For individuals with progressed HCC, palliative systemic therapy is often the only available option. Various tyrosine multikinase inhibitors, such as sorafenib, lenvatinib, regorafenib, and cabozantinib, are approved as first- or second-line treatments for unresectable HCC. Unfortunately, the survival benefits for patients using these treatments are limited [2,3]. The recent introduction of immune-based therapies has created new hope for treating advanced HCC. In particular, the IMbrave150 trial demonstrated that the combination of atezolizumab (an anti-PD-L1 monoclonal antibody) and bevacizumab (a monoclonal antibody targeting VEGF) is superior to sorafenib in terms of overall survival and progression-free survival for advanced HCC patients [4]. However, the benefits of this combination therapy are often temporary, and many patients do not respond to this treatment [4]. As a result, the prognosis for patients with advanced HCC remains poor. Therefore, it is essential to identify novel targets and develop innovative therapies to improve this discouraging scenario.

Growing evidence highlights the critical role of heat shock factor 1 (HSF1) in cancer. HSF1 is the key regulator of the heat shock response (HSR), an essential and evolutionarily conserved defense mechanism that is activated under various stressful conditions [5,6]. Specifically, the HSR protects cells and organisms from the damaging effects of different stressors, including heat, UV radiation, chemicals, hypoxia, and oxidizing agents. When activated, the HSR induces the expression of a specific set of genes and proteins known as heat shock proteins (HSPs), such as HSP90, HSP72, and HSP27. These proteins play vital roles in cellular functions such as protein folding, survival, metabolism, and signaling [5,6]. Given that cancer cells encounter numerous stressful conditions during growth and dissemination, it is not surprising that HSF1 and HSPs are overexpressed in nearly all cancer types. Additionally, HSF1 levels are closely associated with tumor development, invasion, metastasis, and patient prognosis [7,8,9,10,11].

Notably, research has shown that HSF1 contributes to the development and progression of malignant tumors, not only by activating HSPs but also by enhancing oncogene activity, altering metabolism, and suppressing tumor suppressor gene function. Moreover, the genetic depletion or inactivation of HSF1 has been found to inhibit or significantly delay carcinogenesis in several experimental models [12,13,14,15,16,17]. Also considering that HSF1 levels are very low in normal tissues, it represents a promising molecular biomarker and target for cancer treatment [7].

In HCC, previous studies from our laboratory and others have indicated that the levels of HSF1 are significantly linked to tumor initiation, progression, and clinical aggressiveness [13,18,19,20]. To deepen our understanding of the role of HSF1 in liver cancer and to explore innovative treatment options for this type of tumor, we conducted a comprehensive investigation of the antineoplastic activity of NXP800 (CCT361814), a recently developed HSF1 inhibitor currently in clinical trials [21,22,23], using in vitro 2D and 3D HCC models. The present findings indicate that NXP800 exhibits significant antitumor properties against HCC cells, providing a rationale for conducting clinical studies to evaluate the effectiveness of NXP800 in liver cancer patients.

## 2. Results

### 2.1. The HSF1 Inhibitor NXP800 Demonstrates Robust In Vitro Antitumor Activity in HCC Cell Lines

**Figure 1:**
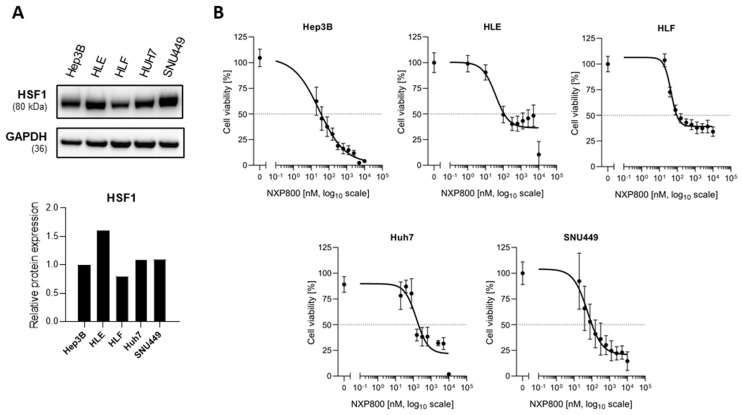
Cytotoxicity assessment of the HSF1 inhibitor NXP800 in human hepatocellular carcinoma (HCC) cell lines. (**A**) The levels of the HSF1 protein were assessed in Hep3B, HLE, HLF, Huh7, and SNU449 cell lines by Western blot analysis. GAPDH was used as a loading control. (**B**) Cell viability was measured via MTT assay. The cell viability in percentage to DMSO control, expressed as non-linear regression curve fit over log10 drug concentrations, is depicted. The mean ± SD (n = 3) of at least three independent experiments each performed in triplicate is shown. Horizontal dotted line: 50% viable cells. IC_50_ values per cell line are shown in each plot.

First, we assessed the levels of HSF1 in a panel of five HCC cell lines (Hep3B, HLE, HLF, Huh7, and SNU449). All HCC cell lines tested expressed the HSF1 protein (Figure 1A). Subsequently, the anti-proliferative properties of NXP800 in vitro were evaluated in the same HCC cell lines using the 3-[4,5-dimethylthiazol-2-yl]-2,5 diphenyl tetrazolium bromide (MTT) assay 48 h after treatment (Figure 1B). Specifically, we tested increasing drug concentrations between 0 and 10 µM, and the half-inhibitory concentration (IC_50_) values were determined using non-linear regression curve fit analyses by GraphPad Prism 10.0.

NXP800 was strongly cytotoxic in the lower nanomolar range against Hep3B (IC_50_ = 31.2 nM), HLE (IC_50_ = 40.6 nM), HLF (IC_50_ = 44.3 nM), Huh7 (IC_50_ = 129 nM), and SNU449 (IC_50_ = 57.5 nM) cell lines (Figure 1B).

**Figure 2:**
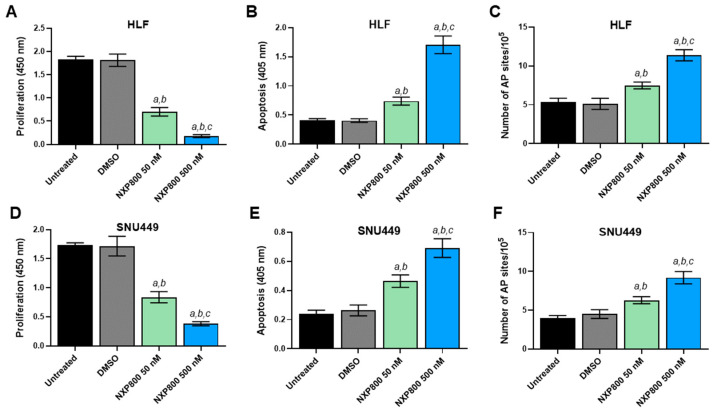
Effects of NXP800 on the proliferation, apoptosis, and DNA damage of HLF and SNU449 human hepatocellular carcinoma cell lines. To evaluate cell proliferation, the BrdU incorporation assay was conducted on HLF (**A**) and SNU449 (**D**) cells treated with NXP800 at concentrations of 50 nM and 500 nM for 48 h. Apoptosis was assessed in HLF (**B**) and SNU449 (**E**) cell lines after treatment with NXP800 for 48 h, using the same concentrations mentioned above. Additionally, a DNA damage assay was performed to measure the formation of apurinic/apyrimidinic (AP) sites, which represent one of the most common types of DNA lesion and a surrogate marker for DNA damage. This assay was applied to both cell lines (**C**,**F**) for 24 h at the two NXP800 concentrations. In all assays, untreated cells and those treated with DMSO (solvent) were used as controls. The results are presented as the mean ± standard deviation (SD) from three independent experiments, each conducted in triplicate. For statistical analysis, Tukey’s multiple comparisons test was used, with significance defined as *p* < 0.001. The following comparisons were conducted: *a* vs. untreated cells; *b* vs. DMSO; *c* vs. 50 nM NXP800.

To further clarify the cellular effects of NXP800, we evaluated proliferation, apoptosis, and DNA damage in the HLF and SNU449 cell lines (Figure 2). Regarding cell proliferation, treatment with NXP800 resulted in a significant decrease in BrdU incorporation in the two cell lines in a dose-dependent manner, with the most pronounced anti-growth effect observed in HLF cells (see Figure 2A,D). Additionally, NXP800 induced apoptosis (Figure 2B,E) and DNA damage (Figure 2C,F) in the two cell lines, with no notable differences found among them. Again, the extent of cell death and DNA damage was higher at higher doses of the drug. Equivalent results on the same parameters were obtained in HLE, Hep3B, and Huh7 cells, with the Hep3B cells showing the most striking decrease in cell proliferation (Supplementary Figure S1). Overall, the current data suggest that NXP800 negatively impacts the growth of HCC cell lines by affecting their proliferation, survival, and DNA integrity.

### 2.2. NXP800 Induces Integrated Stress Response and Modulates Various Signaling and Metabolic Pathways

**Figure 3:**
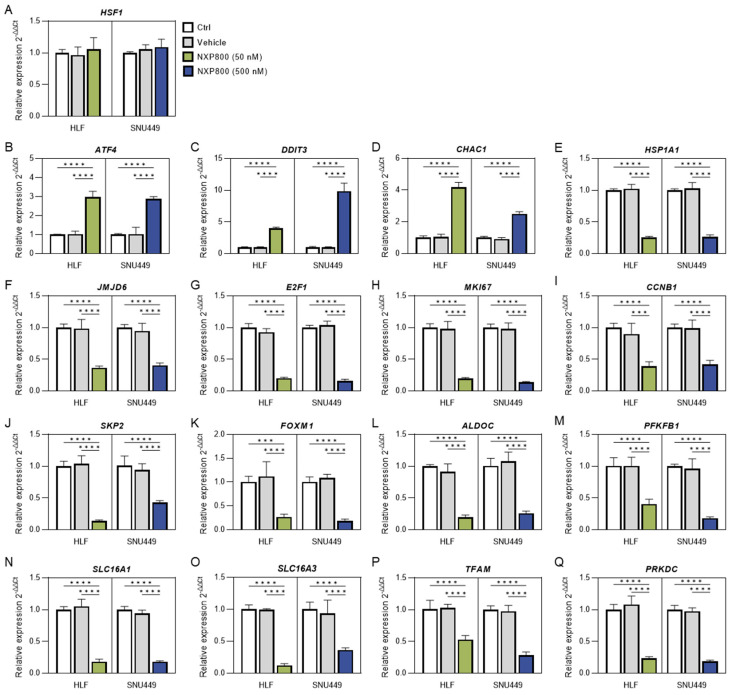
Gene expression analysis in hepatocellular carcinoma cell lines treated with the HSF1 inhibitor NXP800. HLF and SNU449 cells were treated for 48 h with DMSO control (vehicle), or NXP800 (50 nM or 500 nM in HLF or SNU449 cells, respectively). Panels (**A**–**Q**) show the relative mRNA expression levels of the indicated genes, presented as fold change to the untreated control (Ctrl), calculated using the 2^−ΔΔCt^ method, with ACTB used as the housekeeping gene. Data represents the mean ± SD of three independent experiments, each measured in technical duplicates. Statistical analysis was performed using two-way ANOVA followed by Tukey’s multiple comparisons test (*** *p* < 0.001, **** *p* < 0.0001).

Next, the impact of NXP800-mediated functional HSF1 suppression on tumor cell signaling was investigated using quantitative real-time RT-PCR of whole-cell lysates from untreated and NXP800-treated HLF and SNU449 cell lines (Figure 3).

Firstly, we investigated the effect of NXP800 on the levels of integrated stress response (ISR) markers, as it has been shown that this drug induces ISR [22]. The latter is an intracellular signaling transduction network that modulates the response to various stresses [24]. As predicted, we observed a significant increase in *ATF4*, *DDIT3*, and *CHAC1* mRNA levels, consistent with previous data obtained from various cancer cell lines and in vivo mouse models of prostate adenocarcinoma (Figure 3B–D) [22]. In addition, NXP800 administration led to the downregulation of the HSF1 canonical targets *HSPA1A* and *JMJD6* (Figure 3E,F). On the other hand, NXP800 did not affect the mRNA levels of *HSF1* (Figure 3A), in agreement with the notion that this drug interferes with HSF1 activity rather than with its expression [21].

Recently, it has been demonstrated that NXP800 negatively modulates the E2F1 pathway in prostate adenocarcinoma [22]. Thus, we determined whether the same applies to human HCC. Notably, we detected a robust downregulation of *E2F1* and its canonical targets, which are involved in proliferation and promotion of cell cycle progression (*MKI67*, *CCNB1*, *SKP2*, and *FOXM1*), implying that the E2F1 pathway is a critical target of NXP800 and HSF1 in HCC (Figure 3G–K). In light of the established role of HSF1 in regulating glycolysis and mitochondrial integrity [12,25,26], we investigated whether NXP800 affected these metabolic pathways in the same cell lines. Of note, the canonical targets of HSF1 involved in glycolysis, including *ALDOC*, *PFKFB1*, *SLC16A1*, and *SLC16A3*, were all commonly downregulated by NXP800 administration (Figure 3L–O). Similarly, the levels of mitochondrial biogenesis transcription factor *TFAM* were significantly lower in NXP800-treated cells compared to their counterparts treated with DMSO (Figure 3P). Finally, we evaluated the levels of *PRKDC*, the gene encoding the catalytic subunit of the DNA-dependent protein kinase (DNA-PKcs), as we had revealed DNA damage following NXP800 treatment in HCC cells (this study) and had previously identified *PRKDC* as an HSF1 target in HCC [27,28]. Consistent with previous data, *PRKDC* levels were lower in NXP800-treated cells compared to those treated with DMSO (Figure 3Q).

Furthermore, the effect of NXP800 on several oncogenic pathways was investigated by Western blot analysis in HLF and SNU449 cells. No consistent variations in the levels of the mTOR, ERK/MAPK, Stat3, NF-ĸB, and YAP molecular cascades were detected in the two cell lines following the administration of the drug. On the other hand, a slight increase in the AKT and JNK/JUN signaling pathways was induced by NXP800 in both cell lines (Supplementary Figure S2).

Overall, the present data indicate that NXP800 regulates several growth- and metabolic-related pathways.

### 2.3. NXP800 Blocks Glycolysis and Mitochondrial Respiration of HCC Cells

To further substantiate the effects of NXP800 on the glycolytic activity of HCC cells, we utilized the Seahorse XF HS Mini analyzer to perform the glycolytic rate assay on living HLF and SNU449 cells treated with DMSO, 0.3 µM, or 5 µM NXP800 for 24 h. The glycolytic proton efflux rate (glycoPER), determined from the extracellular acidification rate (ECAR), was measured, and key metabolic parameters, including basal glycolysis, compensatory glycolysis, basal PER, and post-2DG acidification, were quantified.

**Figure 4:**
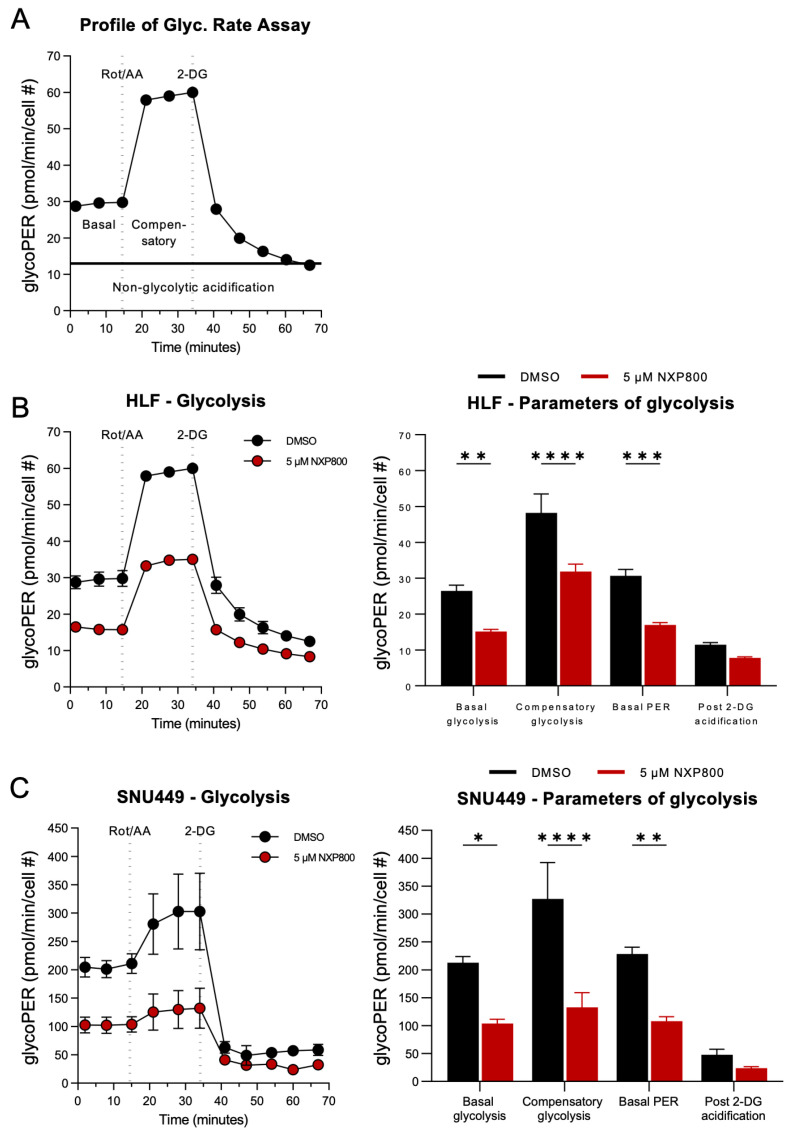
The effect of NXP800 on glycolysis in hepatocellular carcinoma cell lines. HLF and SNU449 cells were treated with DMSO or 5 µM NXP800 for 24 h. Data are presented as mean ± SEM of triplicate samples from two independent experiments, normalized to cell number (#) as quantified by HOECHST 33342 nuclear staining. Values are multiplied by 1 × 10^5^ for visualization purposes. Statistical significance compared to the DMSO control is indicated as * *p* < 0.05, ** *p* < 0.01, *** *p* < 0.001, **** *p* < 0.0001 (two-way ANOVA). (**A**) The standard Glycolytic Rate Assay profile shows the basal and compensatory glycolysis over time. Cells were sequentially challenged with mitochondrial inhibitors rotenone and antimycin A (Rot/AA) to inhibit mitochondrial respiration, which helps reveal the glycolytic proton efflux rate (glycoPER). This was followed by the addition of 2-deoxy-D-glucose (2-DG) to block glycolysis and confirm the specificity of the assay. (**B**,**C**) The glycoPER results (left panel) and bar graphs (right panel) display key parameters such as basal glycolysis, compensatory glycolysis, basal proton efflux rate (PER), and post-2-DG acidification for (**B**) HLF and (**C**) SNU449 cells.

Basal glycolysis, measured prior to Rot/AA injection, was significantly reduced in NXP800-treated cells compared to the DMSO control cells (HLF, ** *p* = 0.0044; SNU449, * *p* = 0.0128; two-way ANOVA; Figure 4A–C). Similarly, compensatory glycolysis, an indicator of the maximal glycolytic capacity of a cell, which was determined after Rot/AA injection, was significantly decreased under NXP800 treatment (HLF, SNU449, **** *p* < 0.0001; Figure 4B,C). Moreover, the basal PER, a measure of proton secretion derived from glycolysis and other minor proton-secreting pathways, was significantly inhibited (HLF, *** *p* = 0.0009; SNU449, ** *p* = 0.0052; Figure 4B,C). The post-2-DG acidification, an indicator of proton secretion from the non-glycolytic origins, showed only slight and statistically non-significant reductions (HLF, *p* = 0.7347; SNU449, *p* = 0.9347; Figure 4B,C). Comparable trends were observed at lower concentrations of 0.3 µM NXP800, as shown in Supplementary Figure S3A,B. These findings suggest that NXP800 administration impairs cellular glycolytic activity, potentially disrupting metabolic homeostasis in HCC cells.

**Figure 5:**
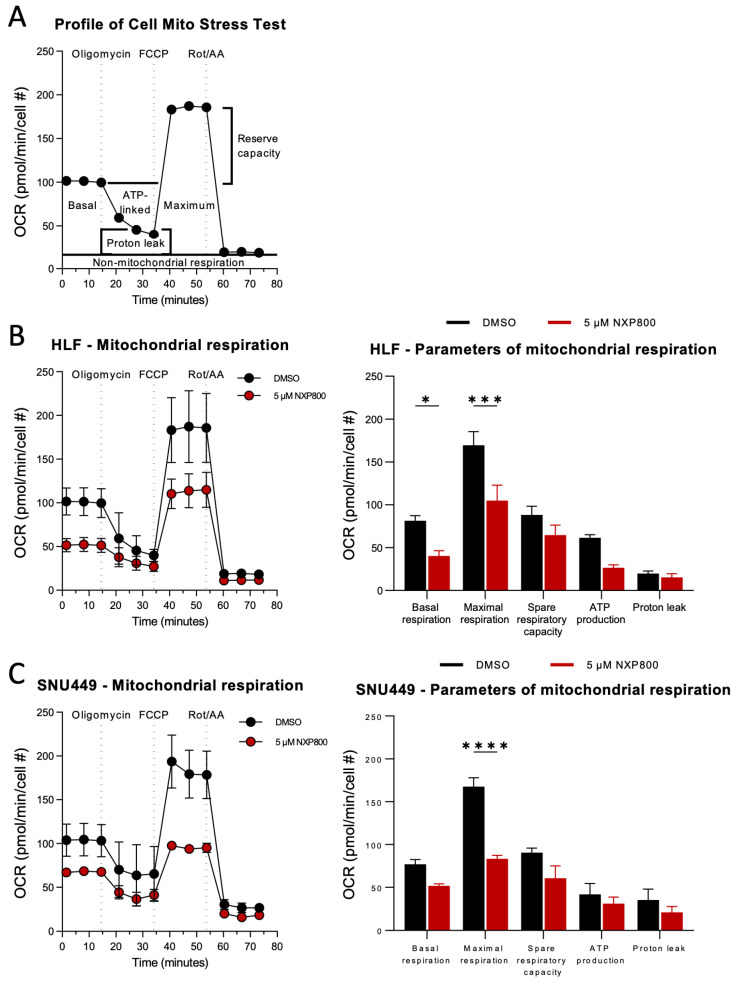
The effect of NXP800 on mitochondrial respiration in hepatocellular carcinoma cell lines. HLF and SNU449 cells were treated with DMSO or 5 µM NXP800 for 24 h. Data are presented as mean ± SEM of triplicate samples from two independent experi-ments, normalized to cell number as quantified by HOECHST 33342 nuclear staining. Values are multi-plied by 1 × 10^5^ for visualization purposes. Statistical significance compared to the DMSO control is indicated as * *p* < 0.05, *** *p* < 0.001, **** *p* < 0.0001 (two-way ANOVA). (**A**) The standard Cell Mitochondrial Stress Test profile shows the oxidative consumption rate (OCR) over time. Basal respiration was followed by sequential injections of oligomycin (which measures ATP-linked OCR), carbonyl cyanide-4-phenylhydrazone (FCCP; which assesses maximal respiration and spare capacity), and Rot/AA (to determine non-mitochondrial OCR). (**B**,**C**) The OCR profile (left panel) and bar graphs (right panel) illustrate basal respiration, maximal respiration, spare respiratory capacity, ATP production, and proton leak for (**B**) HLF and (**C**) SNU449 cells.

In addition, we considered the role of HSF1 pathway signaling for the mitochondrial function of HLF and SNU449 cells as HSF1 signaling is known to be closely tied to mitochondrial function through its role in proteostasis, biogenesis, mitophagy, and metabolic management. Thus, HLF and SNU449 cells were treated with 0.3 µM or 5 µM NXP800 as described above, and mitochondrial respiratory capacity was assessed by measuring the oxidative consumption rate (OCR; Figure 5A–C; Supplementary Figure S3C,D).

The results revealed that NXP800 impaired mitochondrial respiration level as evidenced by a reduction in basal glycolysis (HLF, * *p* = 0.0219; SNU449, *p* = 0.6363), reduced maximal glycolysis (HLF, *** *p* = 0.0002; SNU449, **** *p* < 0.0001), and spare respiratory capacity (HLF, *p* = 0.2718; SNU449, *p* = 0.3874; Figure 5B,C). Furthermore, ATP production and proton leak were measured during oligomycin treatment. Both HLF and SNU449 cells exhibited a mild reduction in ATP production (HLF, *p* = 0.1042; SNU449, *p* = 0.9977) and proton leak (HLF, *p* = 0.9516; SNU449, *p* = 0.9802; Figure 5B,C), suggesting mitochondrial inefficiency and compromised mitochondrial membrane integrity, respectively. Similar but weaker effects were detectable at lower concentrations (Supplementary Figure S3C,D).

In summary, blockage of the HSF1 signaling pathway by NXP800 provokes glycolytic and mitochondrial dysfunction in HLF and SNU449 cells in vitro, potentially due to damage of the inner membrane and disruption of the electron transport chain (ETC).

### 2.4. NXP800 Causes Mitochondrial Injury and Cristae Degradation in HLF Cells

**Figure 6:**
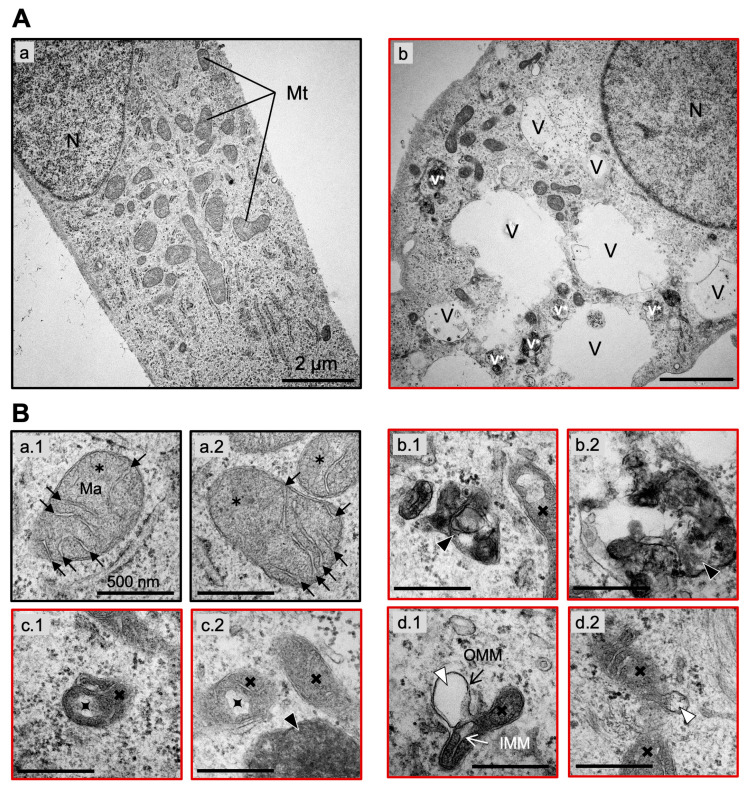
NXP800 causes mitophagy and cristae degradation in HLF cells. (**A**) Representative electron micrographs of (**A**(**a**)) an untreated (DMSO, black outline) and (**A**(**b**)) a 5 µM NXP800-treated (red outline) HLF cell. Mt, mitochondria; N, nucleus; V, electron-lucent vacuoles; v*, electron-dense vacuoles; magnification: 10,000×; scale bars: 2 µm. (**B**(**a**–**d**)) TEM images of two (1 and 2) representative mitochondria of normal (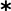) or abnormal (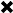) morphology (**B**(**a**): DMSO; **B**(**b**–**d**): 5 µM NXP800). Ma, matrix; IMM, inner mitochondrial membrane; OMM, outer mitochondrial membrane; Black arrow, crista; 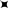, swollen cristae; Black arrowhead, mitophagosome; White arrowhead, ballooning of OMM; Magnification: 40,000×; Scale bars: 500 nm.

To address the extent of mitochondrial damage following exposure to NXP800, mitochondria and cristae morphologies of HLF cells were examined at the ultrastructural level using transmission electron microscopy 48 h after NXP800 (5 µM) administration. DMSO-treated cells were deployed as control, and the mitochondrial phenotype of ten individual tumor cells per group with intact plasma membrane and spherical nucleus (N) was examined (Figure 6A(a,b)).

In untreated HLF cells (DMSO group), mitochondria (Mt) exhibited the “orthodox” state characterized by expanded matrix (Ma) volume of medium electron density and a regular array of few compact and lamellated cristae (black arrows). The inner and outer mitochondrial membranes remained intact with minimal intermembrane spacing (Figure 6A(a); Figure 6B(a)). This condition is considered the energetically efficient configuration of mitochondria under physiological conditions.

Upon treatment with NXP800, significant intracellular and mitochondrial alterations were evident. Most prominently, we observed accumulation of numerous enlarged electron-lucent autophagic vacuoles (V) as well as small electron-dense auto- or mitophagic vacuoles (v*), which were absent in DMSO-treated cells. These findings suggest a severe pathological state induced by NXP800, indicative of mitochondrial stress and active degradation through autophagy and mitophagy (Figure 6A(b)). At a higher magnification (40,000×), two electron-dense mitophagic vacuoles containing degraded mitochondrial components and remnants of other decomposed organelles are shown in greater detail (Figure 6B(b)). These structural changes provide compelling evidence of substantial mitochondrial damage and autophagic activity in response to chemotherapeutic stress induced by NXP800.

Further analysis revealed significant abnormalities in the internal mitochondrial architecture of NXP800-treated HLF cells. Cristae morphologies ranged from bleb-type cristae, as described by Shepard et al. [29], to dilated cristae and in some cases to the complete degeneration of cristae (Figure 6B(c)). Cristae widening and dilation result from severe structural and functional damage to the inner mitochondrial membrane (IMM), which appears as protrusions into the mitochondrial matrix. Such disruption compromises mitochondrial compartmentalization, negatively impacting energy production by oxidative phosphorylation.

In addition to cristae dilation, balloon-like expansions of the outer mitochondrial membrane (OMM) were observed (Figure 6B(d)). Such bulging structures likely reflect mitochondrial dysfunction, contributing to cellular energy deficits, oxidative stress, and eventual cell death.

**Figure 7:**
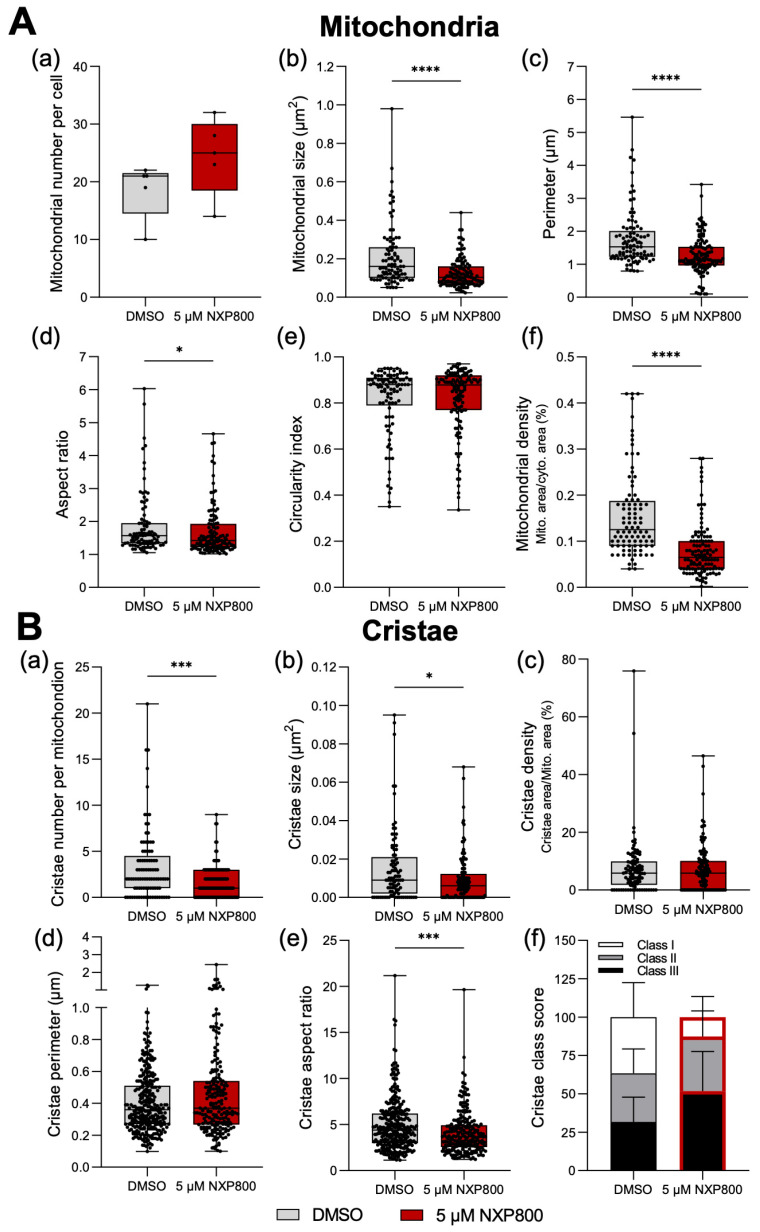
NXP800 causes mitophagy and cristae degradation in HLF cells. (**A**) Quantification of mitochondrial parameters including (**A**(**a**)) mitochondrial number per cell (n = 5 cells; *p* = 0.0873), (**A**(**b**)) size (µm^2^; **** *p* < 0.0001), (**A**(**c**)) perimeter (µm; **** *p* < 0.0001), (**A**(**d**)) aspect ratio (* *p* = 0.0249), and (**A**(**e**) circularity index (*p* = 0.6589), and (**A**(**f**)) density (mitochondrial area in µm/cytoplasmic area in µm, %; **** *p* < 0.0001). The boxplots show min. to max. excluding outliers with median; Mitochondria, n_(DMSO)_ = 93, n_(NXP800)_ = 122. (**B**) Quantification of cristae parameters including (**B**(**a**)) cristae number per mitochondrion (*** *p* = 0.0004), (**B**(**b**)) size (µm^2^; * *p* = 0.0111), (**B**(**c**)) density (cristae area/mitochondria area, %; *p* = 0.6503), (**B**(**d**)) perimeter (µm; *p* = 0.1833), (**B**(**e**)) aspect ratio (*** p = 0.0002), and (**B**(**f**)) cristae class score (class I, >4 cristae per mitochondrion; class II, 2–3 cristae; class III, ≤1 crista). The boxplots show min. to max. excluding outliers with median; Cristae, n_(DMSO)_ = 312, n_(NXP800)_ = 222. Statistical significance is indicated by asterisks (* *p* ≤ 0.05, *** p ≤ 0.001, and **** p ≤ 0.0001; Mann–Whitney test).

For quantitative analysis, mitochondria from five cells per group (n, DMSO = 93; n, NXP800 = 122) were evaluated. While there was no significant difference in the number of mitochondria per cell (Figure 7A(a)), NXP800-treated cells displayed greater variability in mitochondrial counts across cells. This heterogeneity may reflect uneven uptake of NXP800 molecules or differential removal of damaged mitochondria by mitophagy. NXP800-treated also exhibited significantly smaller mitochondria (**** *p* < 0.0001; Figure 7A(b)) with shorter outlines (**** *p* < 0.0001; Figure 7A(c)), and a lower aspect ratio indicating a more stretched shape (* *p* = 0.0249; Figure 7A(d)). However, mitochondrial roundness was comparable between groups (*p* = 0.6589; Figure 7A(e)). Notably, the mitochondria density per cytoplasmic area was significantly reduced in the NXP800-treated cells (**** *p* < 0.0001; Figure 7A(f)), suggesting impaired mitochondrial biogenesis or enhanced degradation.

Analysis of cristae parameters further highlighted the impact of NXP800 treatment on mitochondrial structure. The number of cristae per mitochondrion was significantly reduced (*** *p* = 0.0004; Figure 7B(a)), and cristae size was also diminished (* *p* = 0.0111; Figure 7B(b)). The cristae aspect ratio indicating their shape and organization was significantly lower in NXP800-treated cells (*** *p* = 0.0002; Figure 7B(e)). Conversely, no significant differences were observed in cristae density per mitochondrial area (*p* = 0.6503; Figure 7B(c)) or cristae perimeter (*p* = 0.1833; Figure 7B(d)). However, the cristae score, where class I is defined as >4 cristae per mitochondrion, class II 2–3 cristae and class III ≤ 1 crista, revealed a modest shift in structural integrity (NXP800 class I vs. NXP800 class III, * *p* = 0.0457; Figure 7B(f)).

Collectively, these findings indicate that inhibition of HSF1 signaling in HLF cells by NXP800 induces severe mitochondrial and cristae ultrastructural abnormalities, with profound consequences for mitochondrial health and metabolism. The observed morphological changes align with increased mitophagy and cell death, reflecting a breakdown in mitochondrial integrity and cellular energy production. These ultrastructural defects correlate with functional impairments detected in the Seahorse Cell Mito Stress Test and Glycolysis Rate Assay (Figure 4 and Figure 5), including compromised oxidative phosphorylation capacity and glycolytic collapse.

### 2.5. NXP800 Efficacy in HCC Organoids

**Figure 8:**
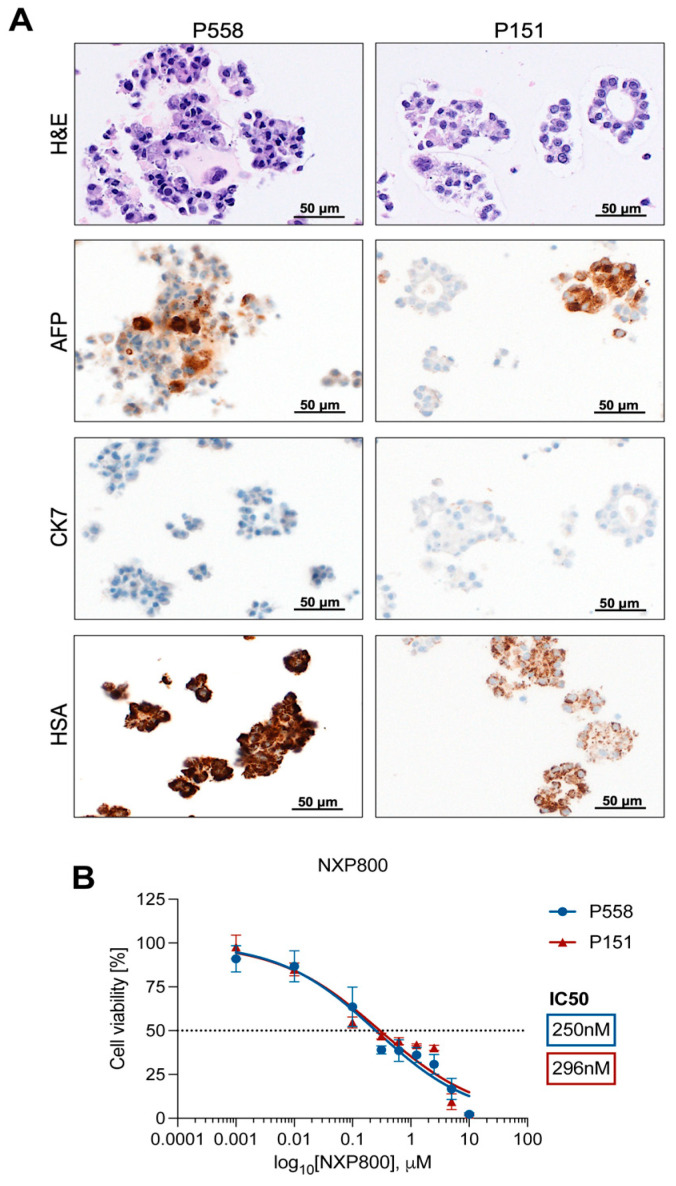
Characterization of patient-derived hepatocellular carcinoma organoids and effect of NXP800 on in vitro growth. (**A**) Characterization of P558 and P151 patient-derived organoids (PDOs). The two organoids exhibited positive immunoreactivity for AFP and HAS hepatocellular markers and were negative for the cholangiocellular marker CK7. Scale bar: 50 µm. Abbreviation: H&E, hematoxylin and eosin staining. (**B**) HCC PDOs were treated for 48 h, and cell viability was evaluated via CellTiter-Glo^®^3D Luminescent Cell Viability Assay (Promega, Madison, WI, USA) according to the manufacturer’s instructions. Cell viability results were normalized on a 0.1% DMSO control, and IC_50_ was obtained on GraphPad Prism 10.0 as a non-linear regression of normalized response.

To further explore the potency of NXP800 in liver cancer, we treated two lines of patient-derived hepatocellular carcinoma (HCC) organoids with NXP800. The P558 and P151 HCC organoids were characterized using hematoxylin and eosin (H&E) staining, as well as immunohistochemistry for the markers alpha-fetoprotein (AFP), cytokeratin 7 (CK7), and hepatocyte-specific antigen (HSA) (Figure 8A). This analysis confirmed the classical HCC morphology of the P558 organoids and the neuroendocrine phenotype of the P151 organoids, consistent with the clinical data from the patients. Next, we determined the IC_50_ of NXP800 for the two different HCC organoid lines, following the previously described methodology for the HCC cell line panel. After 48 h of treatment, both HCC organoid lines demonstrated a similar response trend to the drug, with IC_50_ values of 250 nM for P558 and 296 nM for P151 (Figure 8B).

**Figure 9:**
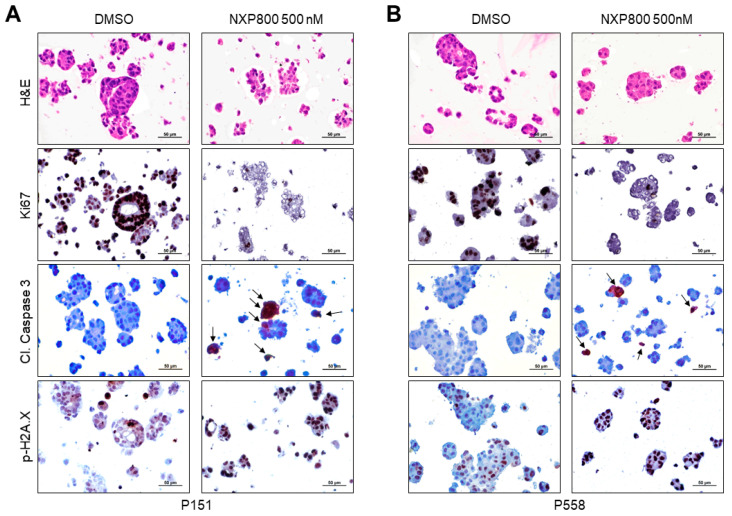
NXP800 treatment induces rarefaction of patient-derived hepatocellular carcinoma organoids, downregulation of the proliferation marker Ki67, and upregulation of apoptosis and DNA damage. The P151 (**A**) and P558 (**B**) patient-derived organoids (PDOs) were treated with 500 Nm NXP800 for 48 h, and levels of the proliferation marker Ki67, the apoptosis marker cleaved caspase 3, and the DNA damage marker phosphorylated/activated (p)histone H2A.X were evaluated immunohistochemically. The two PDOs showed positive immunoreactivity for Ki67 in most of the DMSO-treated PDOs, whereas only a few nuclei were positive for Ki67 immunolabeling in NXP800-treated PDOs. In striking contrast, higher immunoreactivity for cleaved caspase 3 (indicated by arrows) and phosphorylated/activated histone H2A.X characterized the NXP800-treated organoids. Scale bar: 50 µm. Abbreviations: Cl., cleaved; H&E, hematoxylin and eosin staining.

Further treatment with 500 nM NXP800 for an additional 48 h resulted in a significant reduction and rarefaction as well as disruption of organoids in the treated group (Figure 9A,B). Notably, NXP800 treatment led to an almost complete loss of Ki67 immunoreactivity, whereas the DMSO-treated organoids maintained strong Ki67 staining. In addition, the administration of NXP800 resulted in significantly higher levels of apoptosis, as indicated by the presence of cleaved caspase 3, and greater DNA damage, as shown by nuclear immunolabeling for the surrogate marker phosphorylated/activated Histone H2A.X, compared to DMSO in the two organoid models (Figure 9A,B). Therefore, NXP800 not only reduces cell proliferation but also triggers apoptosis and DNA damage in HCC organoids, consistent with our earlier findings in monolayer HCC cell lines.

### 2.6. NXP800 Synergizes with the DNA-Damaging Agent Doxorubicin and the PARP Inhibitor Olaparib to Induce Growth Restraint of HCC Cell Lines

**Figure 10:**
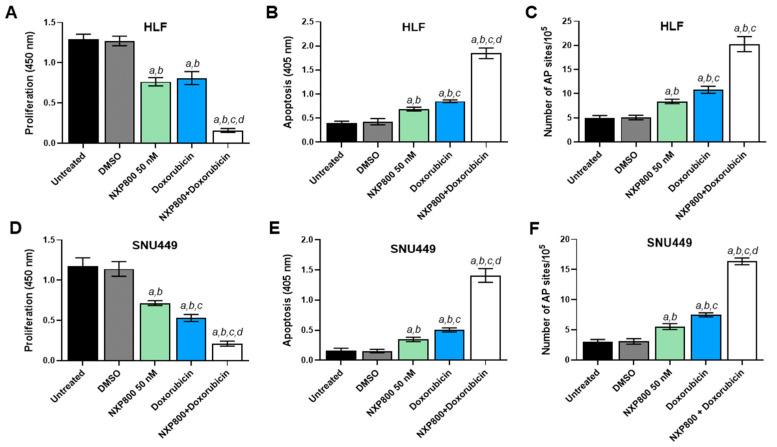
Effect of treatment with NXP800 either alone or associated with doxorubicin administration on cell proliferation, apoptosis, and DNA of HCC cell lines. To evaluate cell proliferation, the BrdU incorporation assay was conducted on HLF (**A**) and SNU449 (**D**) cells treated with NXP800 at a 50 nM concentration, either alone or in association with 50 μM doxorubicin. Apoptosis was assessed in HLF (**B**) and SNU449 (**E**) cell lines after treatment with NXP800 and doxorubicin for 24 h, using the same concentrations mentioned above. Additionally, a DNA damage assay was performed to measure the formation of apurinic/apyrimidinic (AP) sites, which represent one of the most common types of DNA lesion. This assay was applied to both cell lines (**C**,**F**) for 24 h at with NXP800 at a 50 nM concentration, either alone or associated with 50 μM doxorubicin. In all assays, untreated cells and those treated with DMSO (solvent) were used as controls. Treatments were limited to 24 h because massive cell death was observed at 48 h in the HCC cells subjected to the combinatorial treatment. The results are presented as the mean ± standard deviation (SD) from three independent experiments, each conducted in triplicate. For statistical analysis, Tukey’s multiple comparisons test was used, with significance defined as *p* < 0.001. The following comparisons were conducted: *a* vs. untreated cells; *b* vs. DMSO; *c* vs. 50 nM NXP800; *d* vs. doxorubicin.

Finally, we tested a possible combination strategy to enhance the antitumor efficacy of NXP800 in HCC cells. Previous findings indicate that HSF1 plays a critical role in activating DNA-PKcs, which is a key component of the DNA repair machinery [27,28]. Additionally, we discovered that silencing the *PRKDC* gene, which encodes for DNA-PKcs, enhances the effectiveness of the DNA-damaging agent doxorubicin, leading to significant growth reduction in HCC cells [28]. Here, we found that treatment with NXP800 significantly downregulates *PRKDC* and induces DNA damage. Therefore, we investigated whether combining NXP800 with doxorubicin would be more effective than either treatment alone for restraining HCC cell growth. For this purpose, HLF and SNU449 cells were treated with NXP800 alone or following a 2-h pretreatment with a high dose of the carcinogen doxorubicin (50 µM) to induce DNA damage through a single hit (Figure 10). Notably, when NXP800 was administered after the DNA-damaging stimulus, significantly more pronounced reduction in cell proliferation (Figure 10A,D), elevated apoptosis (Figure 10B,E), and massive DNA damage (Figure 10C,F) were observed in the two cell lines compared with either single treatment.

**Figure 11:**
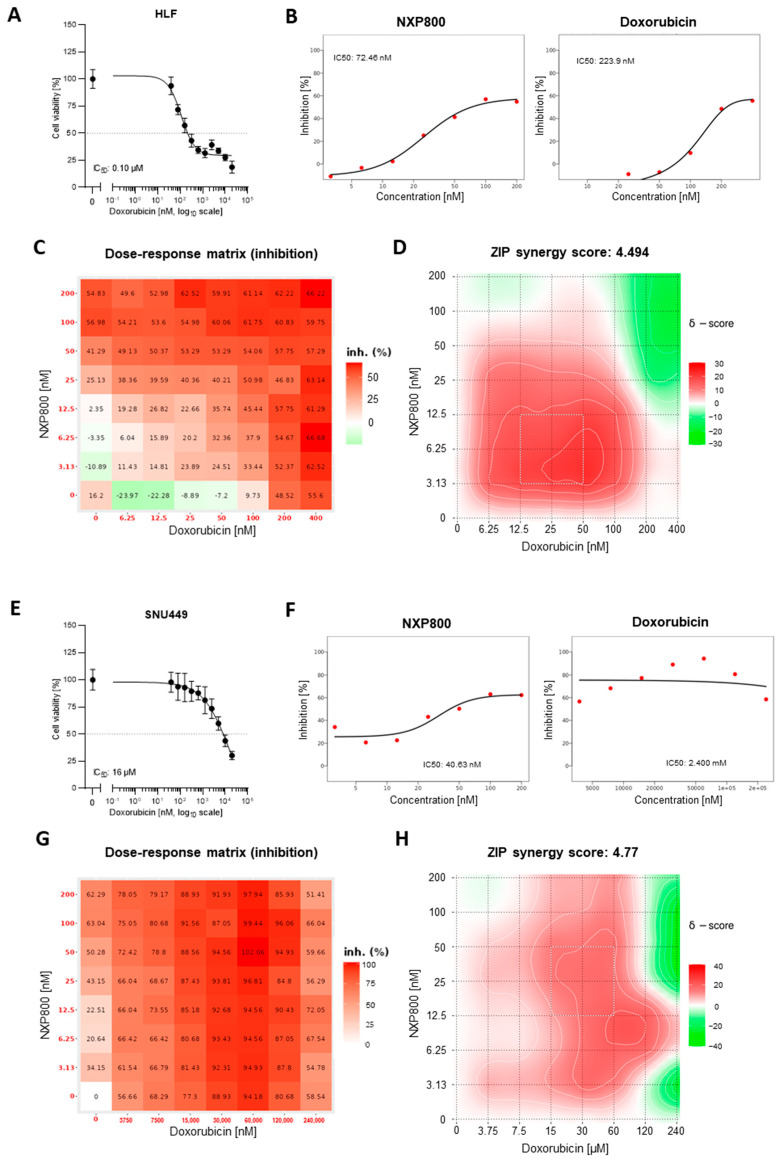
Synergy analysis of NXP800 and doxorubicin in HLF and SNU449 cells. HLF and SNU449 cells were treated with increasing concentrations of NXP800 and doxorubicin as single agents and in combination in a dose–response matrix. Cell viability was assessed by MTT assay after 48 h of treatment, and drug interaction analysis was performed using the ZIP model implemented in SynergyFinder 3. (**A**,**E**) MTT viability assay showing the dose-dependent effect of doxorubicin monotherapy in HLF (**A**) and SNU449 (**E**) cells. (**B**,**F**) Dose–response curves of NXP800 and doxorubicin monotherapies generated using SynergyFinder 3. (**C**,**G**) Inhibition matrices displaying percent growth inhibition as a heatmap (red indicates stronger inhibition). (**D**,**H**) Two-dimensional ZIP synergy score maps for all tested drug combinations. Scores > 5 indicate synergistic interactions.

Next, we determined whether NXP800 and doxorubicin possess synergistic activity against the growth of HCC cells. For this purpose, HLF and SNU449 cells were treated with increasing concentrations of NXP800 combined with escalating doses of doxorubicin (Figure 11). The cell viability was examined by MTT assay 48 h after treatment. Expected drug combination responses were calculated using the Zero Interaction Potency (ZIP) reference model implemented in SynergyFinder 3 (https://synergyfinder.fimm.fi; accessed on 6 March 2026) [30]. First, the sensitivity of both cell lines to doxorubicin monotherapy was determined. As shown in Figure 11A,E, doxorubicin reduced cell viability in a dose-dependent manner in both models. However, SNU449 cells were markedly more resistant than HLF cells, displaying a substantially higher IC_50_ of approximately 16 µM. For synergy analysis, an independent MTT assay was performed in a dose–response matrix design that included increasing concentrations of each compound as monotherapies as well as multiple combination treatments. The resulting dose–response curves illustrate the concentration-dependent effects of NXP800 and doxorubicin as single agents in HLF and SNU449 cells (Figure 11B,F). Consistent with the MTT viability data (Figure 11A,E), both compounds reduced cell viability in a dose-dependent manner, with SNU449 cells showing reduced sensitivity to doxorubicin compared with HLF cells. The inhibition matrices are shown in Figure 11C,G, where the percentage of growth inhibition is displayed in a color-coded heatmap (red indicating stronger inhibition). Due to the pronounced resistance of SNU449 cells to doxorubicin, higher concentrations of doxorubicin were included in the combination matrix for this cell line compared with HLF. The corresponding 2D synergy maps (Figure 11D,H) revealed an overall moderate mean ZIP synergy score for the NXP800–doxorubicin combination in both cell lines. Importantly, distinct regions within the matrices showed strong synergistic interactions, with ZIP synergy scores exceeding 10, indicating concentration-dependent synergistic activity of the two compounds.

**Figure 12:**
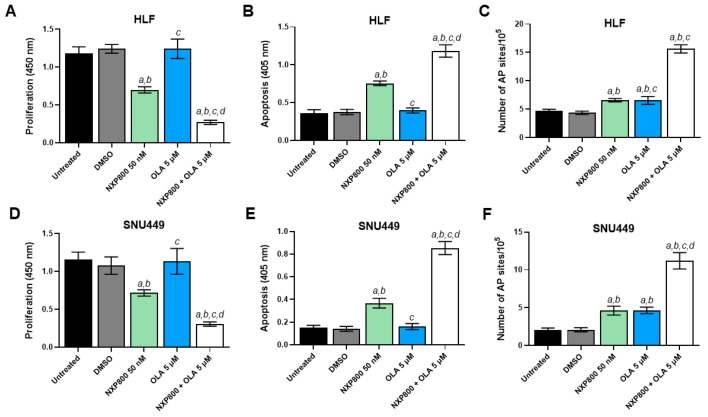
Effect of treatment with NXP800 either alone or associated with the PARP inhibitor olaparib (OLA) on cell proliferation, apoptosis, and DNA of HCC cell lines. To evaluate cell proliferation, the BrdU incorporation assay was conducted on HLF (**A**) and SNU449 (**D**) cells treated with NXP800 at 50 nM concentration, either alone or in association with 5 OLA. Apoptosis was assessed in HLF (**B**) and SNU449 (**E**) cell lines after treatment with NXP800 and OLA, using the same concentrations mentioned above. Additionally, a DNA damage assay was performed to measure the formation of apurinic/apyrimidinic (AP) sites. This assay was applied to both cell lines (**C**,**F**) for 48 h with NXP800 at 50 nM concentration, either alone or associated with 5 μM OLA. Untreated cells and those treated with DMSO (solvent) were used as controls. The results are presented as the mean ± standard deviation (SD) from three independent experiments, each conducted in triplicate. For statistical analysis, Tukey’s multiple comparisons test was used, with significance defined as *p* < 0.001. The following comparisons were performed: *a* vs. untreated cells; *b* vs. DMSO; *c* vs. 50 nM NXP800; *d* vs. OLA.

Recent studies have demonstrated that HSF1 interacts with Poly (ADP-ribose) polymerase 1 (PARP-1) to help cells cope with DNA damage, thereby facilitating DNA repair [31]. Based on this observation, we hypothesize that completely inactivating or disrupting this complex—achieved by simultaneously inhibiting HSF1 using NXP800 and PARP-1 using olaparib (OLA)—could be harmful to the survival of HCC cells. Consequently, we evaluated whether the combination of NXP800 and OLA inhibition limits the growth of HLF and SNU449 cells (see Figure 12). OLA alone did not affect the proliferation or apoptosis of the two cell lines at concentrations up to the maximum tested (50 µM). However, combining OLA at a low concentration of 5 µM with NXP800 significantly decreased proliferation and increased apoptosis and DNA damage compared to NXP800 administered alone (Figure 12).

In summary, the current data suggest that NXP800 may be more effective in treating genetically unstable tumors or when combined with DNA-damaging agents.

## 3. Discussion

Hepatocellular carcinoma (HCC) is the most common type of primary liver cancer and a leading cause of cancer-related deaths worldwide. It is marked by an increasing incidence, late-stage diagnosis, aggressive clinical behavior, and a limited response to existing treatments [1,2,3]. Therefore, finding new targets and therapies for HCC is an urgent and unmet need.

Cumulating evidence underscores the highly relevant function of the multifaceted transcription factor HSF1 in most types of cancer, including HCC, where it contributes to tumor progression, maintenance, and aggressiveness [7,8,9,10,11,12,13,18,19,20]. In particular, it has been shown that genetic deletion of HSF1 or impairment of its transcriptional activity negatively impacts the growth of numerous experimental tumor models, both in vitro and in vivo [12,13,14,15,16,17]. Given the low levels and activity of HSF1 in normal cells, it is considered an attractive target for cancer treatment. However, despite promising results from preclinical models employing genetic approaches, pharmacological attempts to inhibit HSF1 have thus far been unsatisfactory. Many of the HSF1 inhibitors developed in the past have not been optimized for selectivity or desirable drug-like properties. Additionally, there is limited information on their potential mechanisms of action, and they have not yet reached the clinical development stage [32,33]. Therefore, clinical translation of HSF1 inhibitors remains challenging.

Recently, NXP800 (also known as CCT361814), a potent and orally bioavailable fluorobisamide targeting the HSF1 pathway, has been generated [21]. The drug induced profound tumor regression in a human ovarian adenocarcinoma xenograft with on-pathway biomarker modulation and a clean in vitro safety profile [21]. Following its favorable dose prediction to humans, NXP800 entered phase 1 clinical trial (NCT05226507; https://clinicaltrials.gov; accessed on 2 March 2026) in ovarian cancer patients in 2021 as a potential future treatment for refractory ovarian cancer. Subsequently, a phase 1 clinical trial on NXP800 in patients with advanced and metastatic cholangiocarcinoma (NCT06420349) has been started [23].

Here, in the pursuit of developing novel therapeutic approaches for liver cancer, we evaluated the effects of NXP800 on HCC cell growth using HCC cell lines and patient-derived HCC organoids. The present findings reveal that administering NXP800 significantly inhibits the growth of human HCC cell lines and patient-derived organoids, by reducing proliferation, augmenting apoptosis, and triggering DNA damage. The antigrowth effects were achieved by the drug at nanomolar concentrations, confirming previous results obtained in ovarian and prostate cancer [21,22,23].

At the molecular level, the observed reduction in HCC cell proliferation, along with the induction of DNA damage and increased apoptosis, was linked to the downregulation of the E2F1 pathway. This finding aligns with previous reports of NXP800’s inhibitory effects on this molecular cascade in prostate adenocarcinoma models [22]. Furthermore, NXP800 induced the upregulation of the JNK/c-JUN and AKT pathways, suggesting the existence of compensatory mechanisms triggered by HSF1 inhibition. Further studies are necessary to unravel the importance of these signaling cascades in the survival of cancer cells receiving NXP800. Additionally, treatment with NXP800 resulted in a significant decrease in both mitochondrial respiration and glycolysis in HCC cell lines. Notably, NXP800 administration caused severe ultrastructural abnormalities in mitochondria and cristae, compromising mitochondrial integrity and cellular energy production. These data highlight the mitochondria as a key site of NXP800’s activity. We are currently conducting further investigations to identify the specific targets of NXP800 in HCC growth and metabolism.

The promising findings regarding NXP800 present new opportunities for improving treatments for patients with HCC. Because increased HSF1 activity is a general feature of virtually all tumor types, regardless of mutation or transcriptomic profiles [5,6,7,8,9,10,11,12], the present data may have broader implications in cancer, and NXP800 may be a potential treatment for several malignancies. However, it is essential to assess the drug’s effectiveness in preclinical in vivo models before moving on to clinical trials. The data from studies on prostate and ovarian cancer models [21,22] support the drug’s efficacy and tolerability. Additionally, since the single treatment with NXP800 may not be enough to control HCC growth, it will be important to investigate the combination of this drug with other treatment options. In this regard, here we demonstrate that NXP800 works synergistically with the chemotherapeutic drug doxorubicin to restrain HCC growth. We also observed similar results when coupling NXP800 with the PARP inhibitor olaparib. While further research is necessary to assess the effectiveness and safety profile of these combinatory approaches in vivo, combining NXP800 with a DNA-damaging agent could represent a promising new therapeutic option for HCC.

Overall, both previous and current research findings indicate that HCC cells rely heavily on HSF1 activity for their growth, survival, and genome integrity. Therefore, inhibiting HSF1 with NXP800 could be a promising therapy for treating human liver cancer, particularly in conditions that promote genomic instability. This approach could help to improve the landscape of HCC treatment.

## 4. Materials and Methods

### 4.1. Cell Culture

The human hepatocellular carcinoma cell lines HLE (XenoTech, Kansas City, KS, USA; Cat. No. JCRB0404), HLF (Tebubio GmbH, Le Perray en Yvelines, France; Cat. No. JCRB0405), PLC/PRF/5 (CLS Cell Lines Service GmbH, Eppelheim, Germany; Cat. No. 300315), Huh7 (Cytion GmbH, Heidelberg, Germany; Cat. No. 300156), and SNU449 (LGC Standards GmbH, Teddington, Middlesex, UK; Cat. No. CRL-2234) were purchased as indicated and cultured according to standard procedures. Cells were cultured either in Dulbecco’s Modified Eagle Medium (DMEM) or in Roswell Park Memorial Institute (RPMI) 1640 medium supplemented with 10% fetal bovine serum, 100 U/mL penicillin and 100 µg/mL streptomycin, 10 mM HEPES, 1 mM sodium pyruvate, and 2 mM L-glutamine (all from Anprotec, Milpitas, CA, USA) at 37 °C and 5% CO_2_ in a humidified atmosphere. For the induction of DNA damage, cells were treated with 50 μM doxorubicin (Sigma-Aldrich, St Louis, MO, USA) for 2 h, washed twice with phosphate-buffered saline (PBS) and returned to normal growth medium for 24 h.

### 4.2. Chemicals

The HSF1 inhibitor NXP800 (#HY-145927) and the PARP-1 inhibitor olaparib (HY-10162) were purchased from MedChemExpress (Monmouth Junction, NJ, USA). Stock solutions were prepared in 100% dimethyl sulfoxide (DMSO) and were stored at −20 °C. For in vitro experiments, final concentrations were prepared diluting the chemical stocks in fresh culture medium.

### 4.3. Cell Viability Assay (MTT)

Cell cytotoxicity and half-inhibitory concentration (IC_50_) were assessed using the MTT assay. Briefly, cells were seeded into 96-well plates at a density of 1 × 10^4^ cells per well in 100 µL complete medium. After 24 h, fresh medium containing the single drug was added, and the cells were incubated for 48 h at 37 °C and 5% CO_2_. Following the treatment, 10 µL MTT solution (5 mg/mL in PBS) was added to each well and incubated for an additional 2 h at 37 °C protected from light. The supernatant was fully aspired, and the formazan crystals were dissolved in 100 µL DMSO (100%). Formazan absorbances and background absorbances were measured at 570 nm and 630 nm, respectively, in the FLUOstar Omega Microplate Reader (version 5.50 R4, BMG Labtech, Offenburg, Germany) and were analyzed using the MARS data analysis software version 3.32 R5 (BMG Labtech, Offenburg, Germany). The percentage of viable cells was calculated relative to the DMSO control group, the solvent control that was considered 100% viable. All experiments were performed in eight replicates and data of two or three independent experiments were expressed as the mean ± standard error of the mean (SEM). The IC_50_ concentration was determined by non-linear regression analysis in Prism 10.0 (GraphPad Software) using the model (inhibitor) vs. response—Variable slope (four parameters).

### 4.4. Drug Combination Synergy Analysis

Cell viability following drug treatment was assessed using an MTT assay in a dose–response matrix format. Cells were treated with increasing concentrations of NXP800 or doxorubicin alone, as well as with combinations of both compounds arranged in a concentration matrix to evaluate multiple dose combinations. Drug interaction analysis was performed using SynergyFinder 3.0 (https://synergyfinder.fimm.fi) [30]. Within the platform, background correction, automatic outlier detection, and four-parameter log-logistic (LL4) dose–response curve fitting were applied. Synergy scores were calculated using the Zero Interaction Potency (ZIP) model. Two independent biological experiments were performed for each cell line. One representative experiment per cell line is shown.

### 4.5. Western Blot Analysis

Cell pellets were lysed in T-PER^TM^ Tissue Protein Extraction Reagent (Thermo Fisher Scientific, Waltham, MA, USA; 78510) supplemented with 1X Halt^TM^ Phosphatase/Protease inhibitor cocktail (Thermo Fisher Scientific; 78440) and incubated on ice for 1 h. Cell debris was removed by centrifugation at 14,000× *g* for 15 min at 4 °C and total protein was quantified using the Bio-Rad Protein Assay Dye Reagent Concentrate. For SDS-PAGE, equal amounts of protein (5–10 µg) were mixed with 1X Bolt^TM^ Sample Reducing Agent (Thermo Fisher Scientific; B0009) and 1X Bolt^TM^ LDS sample buffer (Thermo Fisher Scientific; B0007) and boiled at 70 °C for 10 min. Proteins were separated on Bolt^TM^ 4 to 12% Bis-Tris Mini Protein Gels (Thermo Fisher Scientific; NW04125BOX) in 1X Bolt^TM^ MES SDS Running Buffer (Thermo Fisher Scientific; B000202) at 110–120 V for 1–2 h. After electrophoresis, proteins were transferred onto nitrocellulose membranes of the iBlot^®^ Gel Transfer Stacks using program 0 (20 V for 1 min, 23 V for 4 min, 25 V for 2 min) the iBLOT 2 Dry Blotting System (Thermo Fisher Scientific; IB21001). Following transfer, membranes were blocked in EveryBlot Blocking Buffer (Bio-Rad, Hercules, CA, USA; 12010020) for 15 min at room temperature. Membranes were then incubated overnight at 4 °C with primary antibodies diluted in blocking buffer. The primary antibodies used at a dilution of 1:1000 were: anti-GAPDH (#2118), anti-HSF1 (#4356), anti-HSP27 (#2402), anti-HSP70 (#4872), anti-HSP90 (#4877), anti-AKT (#9272), anti-phospho-Akt (Ser473; #4060), anti-ERK1/2 (#4695), anti-phospho ERK1/2 (Thr202/Tyr204; #9892), anti-phospho-4EBP1 (Thr37/46; #2855), anti-phospho-RPS6 (Ser235/236; #4858), anti-phospho-Stat3 (Tyr705; #9145), anti-phospho-JNK (Thr183/Tyr185; #4668), anti-c-JUN (#9165), anti-phospho-c-JUN (Ser73; #3270), anti-NF-ĸB p65 (#8242), anti-YAP/TAZ (#14074), and anti-c-Myc (#18583), which were sourced from Cell Signaling Technology (CST, MA, USA). The following day, the membranes were washed three times with 1X TBS buffer (CST, Cat. #9997) and incubated with the appropriate horseradish peroxidase (HRP)-conjugated secondary antibody (1:20,000; Abbkine; A21010, A21020) for 1 h at room temperature. After washing the membranes three times with 1X TBS buffer, the protein bands were detected using the Clarity Max ECL Western Blotting Substrate (Bio-Rad) and visualized with the ChemiDoc MP (Bio-Rad; 17001402). Uncropped images of the Western Blots are depicted in Supplementary Figure S4).

### 4.6. Quantitative Real-Time RT-PCR (qRT-PCR)

Total mRNA was extracted from HLF and SNU449 HCC cell lines with the Quick RNA Miniprep kit (Zymo Research, Irvine, CA, USA). Subsequently, mRNA expression of the genes of interest was assessed by qRT-PCR. The following, human-specific TaqMan Gene Expression Assays (Thermo Fisher Scientific) were used: *HSPA1A* (Hs00359163_s1), *JMJD6* (Hs00397095_m1), *HSF1* (Hs01027616_g1), *E2F1* (Hs00153451_m1), *MKI67* (Hs04260396_g1), *Cyclin B1* (*CCNB1*; Hs01030099_m1), *SKP2* (Hs01021864_m1), *FOXM1* (Hs01073586_m1), *ALDOC* (Hs00902799_g1), *PFKFB1* (Hs00997227_m1), *SLC16A1* (Hs01560299_m1), *SLC16A3* (Hs00358829_m1), *TFAM* (Hs00273372_s1), and *PRKDC* (Hs04195439_s1). PCR reactions were conducted using 100 ng of cDNA from the collected samples, with an ABI Prism 7000 Sequence Detection System with TaqMan Universal PCR Master Mix (Applied Biosystems, Waltham, MA, USA). Cycling conditions were as follows: denaturation at 95 °C for 10 min, 40 cycles at 95 °C for 15 s, and then extension at 60 °C for 1 min. Quantitative values were calculated using the PE Biosystems Analysis software version 1.2.3 and expressed as N target (NT). NT  =  2^−ΔCt^, where each sample’s ΔCt value was calculated by subtracting the average Ct value of the target gene from the average Ct value of the *β-actin* gene (4333762T; Thermo Fisher Scientific).

### 4.7. Seahorse Mitochondrial Respiration and Glycolysis Analyses

The effect of NXP800 on mitochondrial function and glycolytic capacity of HLF cells was assessed using the Seahorse XF Mito Stress Test Kit and Seahorse XF Glycolytic Rate Assay Kit (Agilent Technologies, Santa Clara, CA, USA) according to the manufacturer’s protocol. Optimized cell density and carbonyl cyanide-4 phenylhydrazone (FCCP) concentration for HLF cells were determined. For both assays, 4.0 × 10^4^ cells per well were seeded in 80 µL complete culture medium in Seahorse XFp 8 well cell culture plates and cultured overnight at 37 °C and 5% CO_2_ in a humidified atmosphere. A sensor cartridge was hydrated in Seahorse XF Calibrant at 37 °C in a non-CO_2_ incubator overnight as well. On the day of the assays, the culture medium was replaced by Seahorse XF DMEM (pH 7.4) supplemented with 1 mM sodium pyruvate, 2 mM glutamine, and 10 mM glucose. The cells were incubated in a non-CO_2_ incubator at 37 °C for 1 h to equilibrate. For the Mito Stress Test, the oxygen consumption rate (OCR; pmol/min), an indicator for oxidative phosphorylation, was measured before and after the serial injection of oligomycin (1 µM final concentration), FCCP (2 µM) and rotenone/antimycin A (0.5 µM each) using the Seahorse XF HS mini analyzer (Agilent Technologies, Santa Clara, CA, USA). Basal and maximal respiration, spare respiratory capacity, ATP production, and proton leak were calculated based on the OCR data. For the Glycolytic Rate Assay, OCR and the extracellular acidification rate (ECAR), an indicator for glycolysis, were measured to determine the glycolytic proton efflux rate (glycoPER) at multiple time points before and after serial injection of rotenone/antimycin A (0.5 µM each) and 2-deoxy-d-glucose (50 mM). Basal and compensatory glycolysis, basal proton efflux rate, and post 2-DG acidification were calculated based on the glycoPER data. Data of both assays were normalized to the cell number per well determined by Hoechst 33342 (0.002 µg/mL; Thermo Fisher Scientific) nuclear staining. After completing the Seahorse assays, cells were stained with Hoechst 33342 dye for 10 min at room temperature and washed twice with PBS. The fluorescence was measured at an excitation of 355-20 nm and emission of 520 nm using the FLUOStar Omega plate reader. Statistical significance was determined using a two-way ANOVA.

### 4.8. Transmission Electron Microscopy (TEM)

HLF cells were grown at a density of 0.3 × 10^6^ cells per 6 well and exposed to DMSO and 5 µM NXP800 for 48 h. Cells were fixed in 0.1 M cocadylate buffer supplemented with 4% glutaraldehyde overnight at room temperature in a fume hood. The samples were pelleted, resuspended in Epredia Cytoblock Cell Block Preparation System (Thermo Fisher Scientific), and surrounded by 4% low melting agarose. For the embedding process including post-fixation with 1% osmium tetroxide, dehydration in 30–100% ethanol, and infiltration with EPON resin, the LYNX microscopy tissue processor (Reichert-Jung, Wetzlar, Germany) was utilized. Semi-thin sections (0.75 µm) for the selection of relevant areas and ultra-thin sections (80 nm) were cut using the Ultracut S Ultramicrotome (Leica-Reichert, Wetzlar, Germany) and picked up on grids. The sections were contrasted with aqueous 2% uranyl-acetate followed by 2% lead-citrate for 10 min each. High resolution imaging was performed using the LEO 912AB transmission electron microscope (Zeiss, Oberkochen, Germany) operating at 100 kV.

For analysis, mitochondria of five cells per group were imaged at 10,000× and 40,000× magnification. TEM images were then uploaded to ImageJ (version 1.54f, NIH) to quantify diverse parameters using Fiji Plugin image processing tool: Mitochondrial number per cell, area, perimeter, aspect ratio and circularity index. In addition, cristae parameters including number per mitochondrion, area, perimeter and aspect ratio were evaluated. Based on the cristae number per mitochondrion, a cristae score was defined: class I (>4 cristae per mitochondrion), class II (2–3 cristae) and class III (≤1 crista). Significances were calculated using the Mann–Whitney test.

### 4.9. HCC Organoids

Patient-derived organoids (PDOs) lines used in this study were derived from HCC tissue collected from two patients undergoing surgery in the University Center for Gastrointestinal and Liver Disease (Clarunis), Basel (Switzerland), under written informed consent, in accordance with the Helsinki Declaration and approved by the Ethics Committee of Basel (EKBB, no 2019-02118) as described previously [34,35]. Briefly, HCC samples were collected in Ad-DF+++ medium, (Advanced DMEM/F12 (Gibco), supplemented with 1% Glutamax^TM^, HEPES (Thermo Fisher), Pen-Strep (Gibco, Waltham, MA, USA), and 100 μg/mL primocin (InvivoGen, San Diego, CA, USA), minced into 1–2 mm^3^ fragments with a scalpel and enzymatically dissociated for 1 h at 37 °C under constant rotation in a Miltenyi Biotec MACSmix Tube Rotator in Dissociation Buffer (*Ad-DF+++* medium supplemented with 2.4 mg/mL Collagenase IV (Worthington); 7.2 μg/mL DNase (Sigma-Aldrich, St. Louis, MO, USA), 270 μg/mL Hyaluronidase (Sigma-Aldrich), 0.1%BSA (Sigma-Aldrich), 20 μM Rock Inhibitor (Y-27632 dihydrochloride, Abmole Bioscence, Houston, TX, USA)). Enzymatic dissociation was stopped with 1%FBS (Sigma-Aldrich), and the dissociation suspension was filtered through a 100 μM cell strainer in a falcon tube and centrifuged at 300× *g* for 10 min at 8 °C. Pellet was resuspended and incubated for 3 min at room temperature in Red Blood Lysis buffer (Roche, Basel, Switzerland) to remove red blood cells, followed by a washing step with *DPBS 1X* and a second centrifugation. Finally, pellet was resuspended in Ad-DF+++ for cell counting in a Countess^TM^ Automated Cell Counter (Thermo Fisher) with trypan blue in 1:2 dilution. Cells were resuspended in ice in an appropriate volume of Matrigel^®^ (Corning, Growth Factor Reduced (GFR) Basement Membrane Matrix) to plate 1 × 10^6^ cells/well in pre-warmed six-well plates in 20 μL Matrigel^®^ domes, and placed inverted at 37 °C for 20 min. Finally, HCC medium (Ad-DF+++ supplemented with 1xN2 (Gibco), 1XB27 (B27^TM^ Supplement 50X serum free, Thermo Fisher), 10 mM Nicotinamide (NIC, Sigma-Aldrich), 1.25 mM N-acetyl-l-cysteine (NAC, Sigma-Aldrich), 5 μM A83-01 (PeproTech, Cranbury, NJ, USA), 50 ng/mL Human epidermal growth factor (EGF, PeproTech), 100 ng/mL FGF10 (Novus Biologicals, Littleton, CO, USA), 500 ng/mL R-spondin 1 protein (EPFL, Lausanne, Switzerland), 10 μM Forskolin (Tocris, Bristol, UK), 25 ng Hepatocytes Growth Factor (HGF, PeproTech), 10 nM Gastrin (Sigma-Aldrich)) was added to allow organoid growth. HCC organoids were passaged every 3–5 days according to the PDOs growth rate at 1:3/4 split rate. Briefly, HCC medium was removed, Matrigel^®^ domes were detached with a scraper and collected in 700 μL TrypLE^®^ Express 1X—Phenol Red (Gibco) per well in a 15 mL falcon tubes. Enzymatic dissociation was performed at 37 °C in a water bath for up to 15 min, including 3–4 pipetting steps. Organoid dissociation was checked under a light microscope until dissociation to single cells, stopped with Stopping Medium (DMEM, 10% FBS, 1% Pen-Strep), and falcon tubes were centrifuged for 5 min at 300 g at 4 °C. Supernatant was discarded and pellet was resuspended in an appropriate volume of Matrigel^®^ or BME type II (Cultrex Basement Membrane Extract, Type 2, Pathclear, R&D Systems, Minneapolis, MN, USA) to make the domes as previously described.

### 4.10. HCC Organoids Drug Screening

For drug screening, HCC organoids were seeded as single cells in a layer of BME in 384-well plates. Briefly, 10 μL of BME:HCC medium at 1:1 ratio were added in each well of a pre-cooled 384-well plate, centrifuged at +4 °C and placed at 37 °C for 1 h. Once reached confluency, HCC PDOs were collected as in the passaging procedure. Following the enzymatic dissociation step, HCC PDOs were resuspended in Stopping Medium and filtered through 40 μm cell strainer, washed with cold *DPBS 1X* and centrifuged at 300× *g* for 5 min at 4 °C. Pellet was resuspended in HCC medium for automatic cell counting: 5000 cells/well were plated manually in the prepared 384-well plate, centrifuged, and placed in the incubator overnight. The day after, 9 different drug concentrations of NXP800 (MedChemExpress, Monmouth Junction, NJ, USA), including 1 nM-10 nM-100 nM and 0,313-10 μM at 1:2 dilution were loaded with the automatic dispenser TecanD300e in the 384-well plate. Positive and negative controls of 2 μM Staurosporine and 0.1%DMSO, respectively, were included in the assay. HCC PDOs were treated for 48 h and cell viability was evaluated via CellTiter-Glo^®^3D Luminescent Cell Viability Assay (Promega) according to the manufacturer instructions. Luminescence was recorded at Microplate Reader RE 6.0.2.3 (Thermo Fisher) with Skanlt Software 6.0.2. Cell viability results were normalized on 0.1%DMSO control, and EC_50_ was obtained on GraphPad Prism 10.0 as non-linear regression of normalized response.

### 4.11. HCC Organoids FFPE

HCC PDOs were formalin-fixed and paraffin-embedded (FFPE) as previously described [34,35]. Overall, 4 μm of FFPE slides was cut with a microtome and stained for H&E and immunohistochemistry of anti-HSA (Roche, #760-4350), anti-AFP (Roche, #760-2603), and anti-CK7 (Roche, #790-4462) markers for organoid characterization. Pathological evaluation confirmed HCC features in both PDOs, P558 as classic HCC while P151 a neuroendocrine HCC type. For NXP800 treatment, HCC PDOs were grown for 4 days and treated with 500 nM NXP800 for 48 h. Medium was removed, and HCC PDOs were collected and included in FFPE blocks as described. To assess proliferation, apoptosis, and DNA damage in the organoids, the following antibodies at 1:400 dilution were applied: anti-Ki67 (#9449), anti-cleaved caspase 3 (#9661), and anti-phospho-histone H2A.X (Ser139; #9718), from Cell Signaling Technology.

### 4.12. Statistical Analysis

Data is presented as the mean ± standard error of the mean (SEM) determined from two or more experiments. Statistical significance and IC_50_ values were determined using the software Prism 10.0. A *p*-value of <0.05 was considered statistically significant. Significances were calculated using a two-way ANOVA (Seahorse) for multiple comparison and unpaired Mann–Whitney test (TEM analysis). IC_50_ values were estimated via non-linear regression curve-fit, applying the “(inhibitor) vs. normalized response—variable slope” model.

## Data Availability

All data generated or analyzed during this study are included in this published article (and its Supplementary Information Files).

## Supplementary Materials

The following supporting information can be downloaded at: https://www.mdpi.com/article/10.3390/ijms27062781/s1.

## Abbreviations

The following abbreviations are used in this manuscript:

AFP, Alpha-fetoprotein; ALDOC, Aldolase C; ATF4, Activating transcription factor 4; BME, Basement membrane extract; BrdU, Bromodeoxyuridine; CCNB1, Cyclin B1; CHAC1, Glutathione specific gamma-glutamylcyclotransferase 1; CK7, Cytokeratin 7; DDIT3, DNA damage inducible transcript 3; 2-DG, 2-deoxy-D-glucose; DMEM, Dulbecco’s Modified Eagle Medium; DMSO, Dimethyl Sulfoxide; DNA, Deoxyribonucleic acid; E2F1, E2F Transcription factor 1; ECAR, extracellular acidification rate; EGF, Human epidermal growth factor; ETC, Electron transport chain; FCCP, Carbonyl cyanide-4-phenylhydrazone; FFPE, Formalin-fixed, paraffin-embedded; FOXM1, Forkhead box M1; GAPDH, Glyceraldehyde-3-phosphate dehydrogenase; GFR, Growth factor reduced; glycoPER, glycolytic proton efflux rate; HCC, Hepatocellular carcinoma; H&E, Hematoxylin and eosin; HEPES, 2-[4-(2-hydroxyethyl)piperazin-1-yl]ethanesulfonic acid; HGF, Hepatocytes growth factor; HRP, Horseradish peroxidase; HSA, Hepatocyte specific antigen; HSF1, Heat-shock factor 1; HSP, Heat Shock Protein; HSPA1A, Heat Shock Protein Family A (Hsp70) Member 1A; HSR, Heat-shock response; IC_50_, Half-inhibitory concentration; IHC, Immunohistochemistry; IMM, Inner mitochondrial membrane; ISR, Integrated stress response; LDS, Lithium dodecyl sulfate; NAC, N-acetyl-l-cysteine; NIC, Nicotinamide; OCR, Oxidative consumption rate; OMM, Outer mitochondrial membrane; PARP-1, Poly (ADP-ribose) polymerase 1; PBS, Phosphate-buffered saline; PD-L1, Programmed cell death Ligand 1; PDO, patient derived organoid; PFKFB1, 6-Phosphofructo-2-kinase/fructose-2,6-biphosphatase 1; PRKDC, Protein kinase, DNA-activated, catalytic subunit; qRT-PCR, Quantitative reverse transcription-polymerase chain reaction; Rot/AA, Rotenone/Antimycin A; RPMI 1640, Roswell Park Memorial Institute 1640; SD, Standard deviation; SDS-PAGE, Sodium dodecyl sulfate-poly acrylamide gel electrophoresis; SKP2, S-phase kinase associated protein 2; TFAM, Transcription factor A, mitochondrial; UV, Ultraviolet; VEGF, Vascular Endothelial Growth Factor.
