# Supplementary material for: The Novel HSF1 Inhibitor NXP800 Exhibits Robust Antitumor Activity in Hepatocellular Carcinoma
Source: Int J Mol Sci. 2026 Mar 19;27(6):2781. doi: 10.3390/ijms27062781 (PMC13026560; doi:10.3390/ijms27062781)
Supplement: Supplementary file 1 [file ijms-27-02781-s001.zip › ijms-4078062-supplementary.pdf]

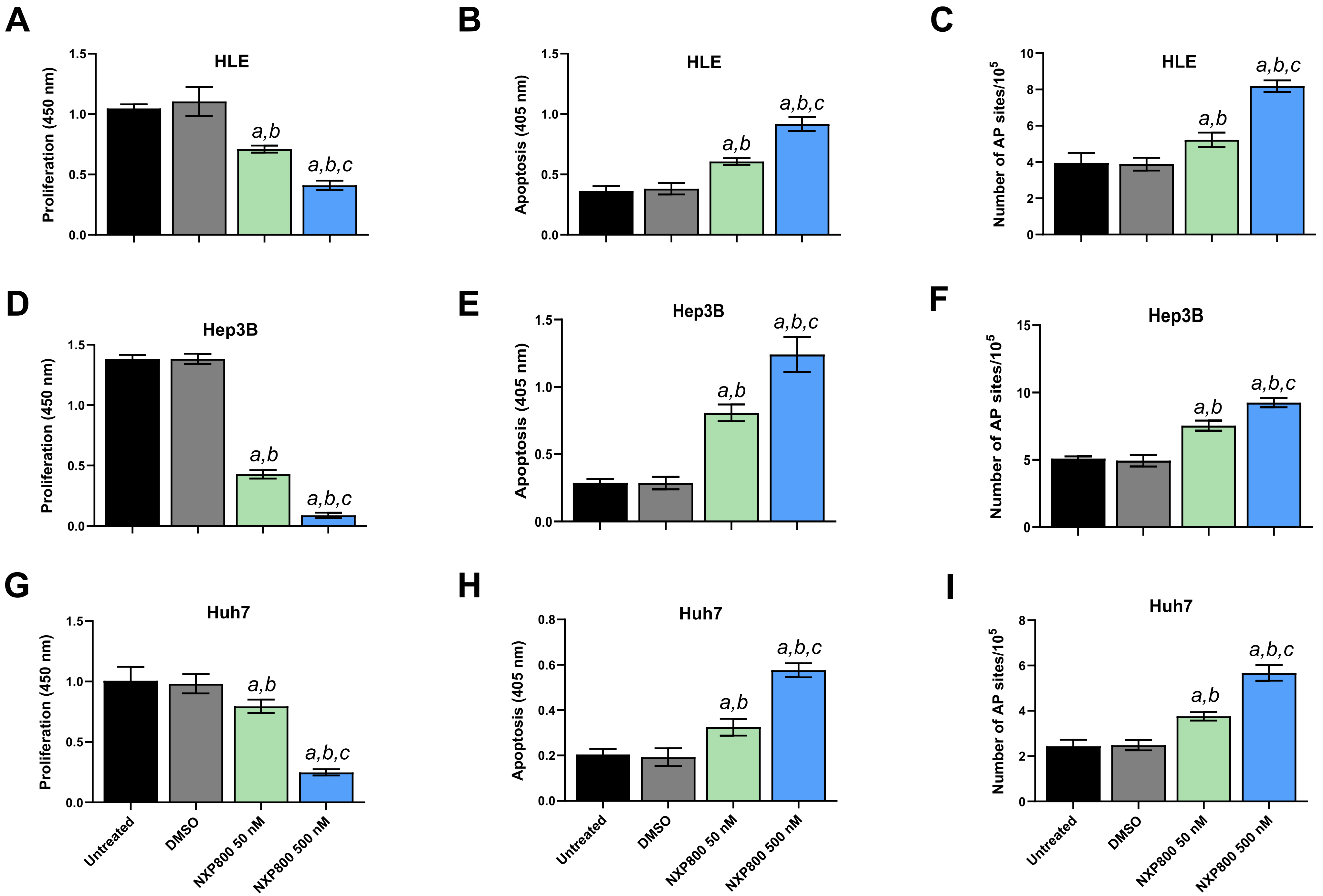

A
HLE
Proliferation (450 nm)
a,b
a,b,c
B
HLE
Apoptosis (405 nm)
a,b
a,b,c
C
HLE
Number of AP sites/10^5
a,b
a,b,c
D
Hep3B
Proliferation (450 nm)
a,b
a,b,c
E
Hep3B
Apoptosis (405 nm)
a,b
a,b,c
F
Hep3B
Number of AP sites/10^5
a,b
a,b,c
G
Huh7
Proliferation (450 nm)
a,b
a,b,c
H
Huh7
Apoptosis (405 nm)
a,b
a,b,c
I
Huh7
Number of AP sites/10^5
a,b
a,b,c
Untreated
DMSO
NXP800 50 nM
NXP800 500 nM

Supplementary Figure S1. Effects of NXP800 on the proliferation, apoptosis, and DNA damage of HLE, Hep3B, and Huh7 human hepatocellular carcinoma cell lines. To evaluate cell proliferation, the BrdU incorporation assay was conducted on HLE (A), Hep3B (D), and Huh7 (G) cells treated with NXP800 at concentrations of 50 nM and 500 nM for 48 hours. Apoptosis was assessed in HLE (B), Hep3B (E) and Huh7 (H) cell lines after treatment with NXP800 for 48 hours, using the same concentrations mentioned above. Additionally, a DNA damage assay was performed to measure the formation of apurinic/apyrimidinic (AP) sites, which represent one of the most common types of DNA lesions and a surrogate marker for DNA damage. This assay was applied to the same cell lines (C, F, and I) for 24 hours at the two NXP800 concentrations. In all assays, untreated cells and those treated with DMSO (solvent) were used as controls. The results are presented as the mean ± standard deviation (SD) from three independent experiments, each conducted in triplicate. For statistical analysis, Tukey's multiple comparisons test was used, with significance defined as $p < 0.001$. The following comparisons were performed: a vs. untreated cells; b vs. DMSO; c vs. 50 nM NXP800.

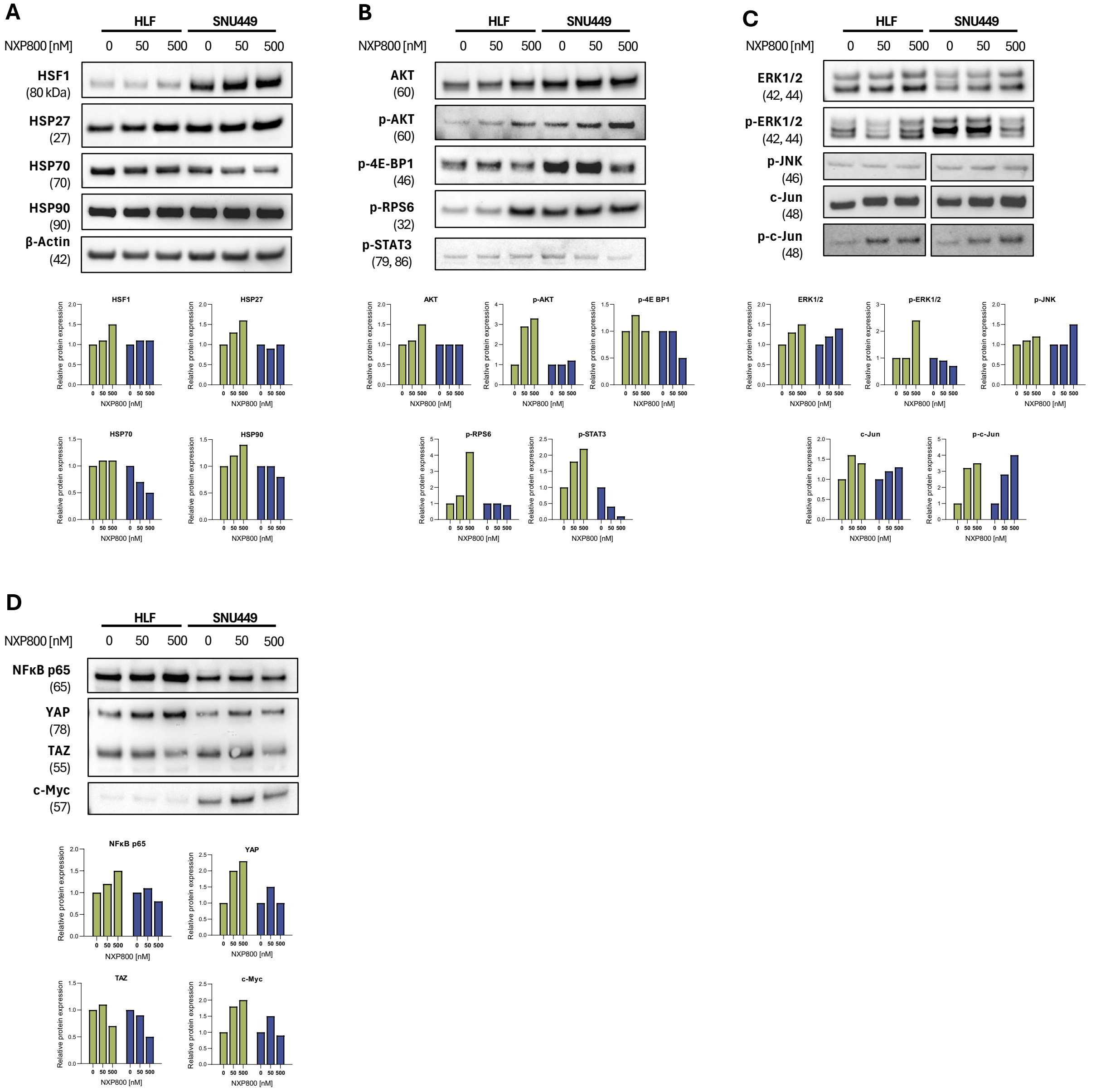

Supplementary Figure S2. Western Blot analysis of the effects of the HSF1 inhibitor NXP800 on oncogenic cascades in HLF and SNU449 HCC cell lines. (A-D) Representative Western blot membranes showing the expression of the indicated proteins are displayed alongside the corresponding densitometric quantification presented as bar graphs. Protein levels were normalized to β-actin (A) and expressed relative to the control condition (0 nM NXP800).

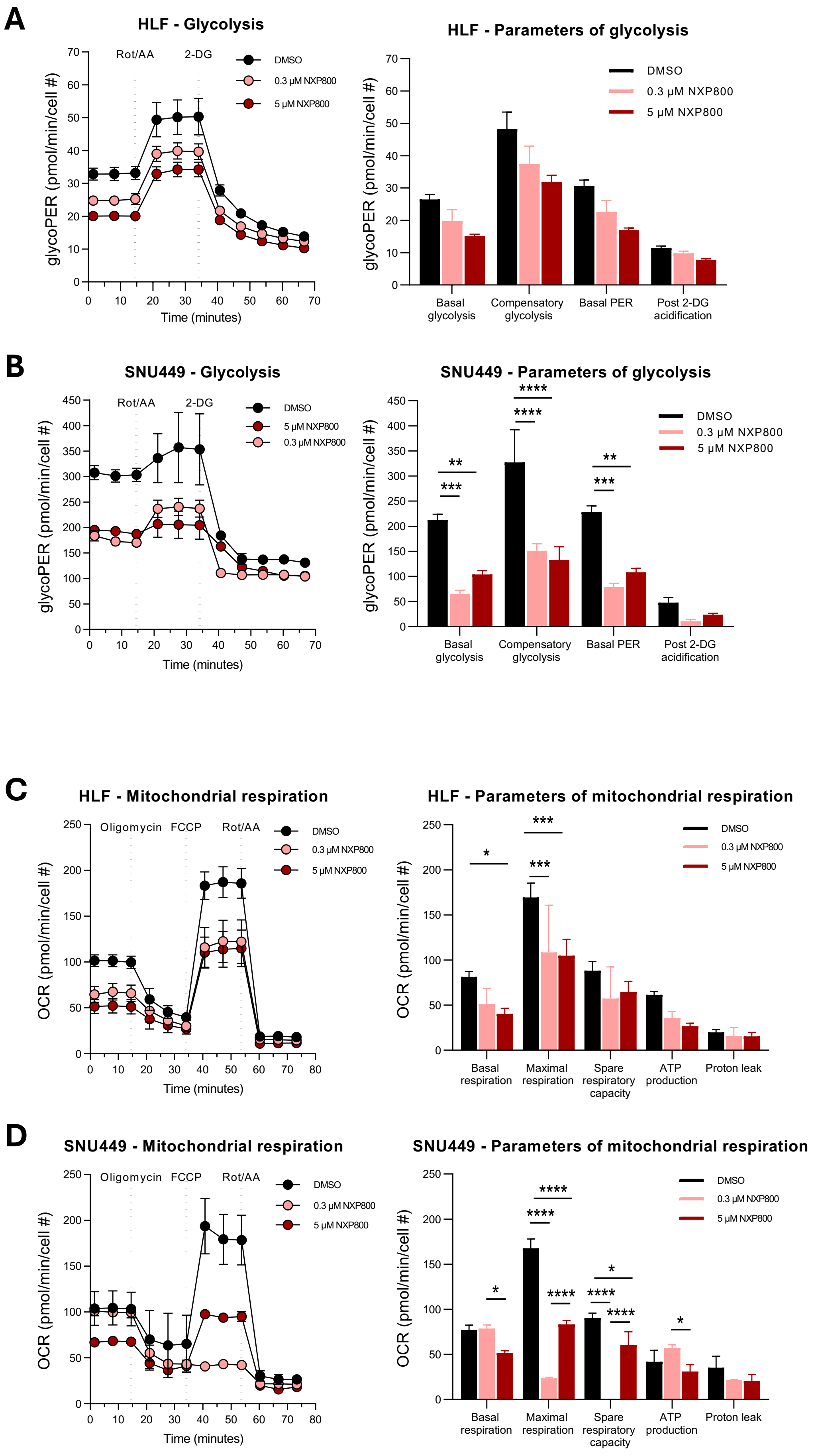
A
HLF - Glycolysis
Rot/AA
2-DG
DMSO
0.3 µM NXP800
5 µM NXP800
glycoPER (pmol/min/cell #)
Time (minutes)
HLF - Parameters of glycolysis
glycoPER (pmol/min/cell #)
Basal glycolysis
Compensatory glycolysis
Basal PER
Post 2-DG acidification
B
SNU449 - Glycolysis
Rot/AA
2-DG
DMSO
5 µM NXP800
0.3 µM NXP800
glycoPER (pmol/min/cell #)
Time (minutes)
SNU449 - Parameters of glycolysis
**
***
****
****
**
***
Basal glycolysis
Compensatory glycolysis
Basal PER
Post 2-DG acidification
C
HLF - Mitochondrial respiration
Oligomycin
FCCP
Rot/AA
DMSO
0.3 µM NXP800
5 µM NXP800
OCR (pmol/min/cell #)
Time (minutes)
HLF - Parameters of mitochondrial respiration
*
***
***
Basal respiration
Maximal respiration
Spare respiratory capacity
ATP production
Proton leak
D
SNU449 - Mitochondrial respiration
Oligomycin
FCCP
Rot/AA
DMSO
0.3 µM NXP800
5 µM NXP800
OCR (pmol/min/cell #)
Time (minutes)
SNU449 - Parameters of mitochondrial respiration
*
****
****
****
*
****
****
*
Basal respiration
Maximal respiration
Spare respiratory capacity
ATP production
Proton leak

Supplementary Figure S3. Effect of NXP800 on glycolysis and mitochondrial respiration in hepatocellular carcinoma cell lines. HLF and SNU449 cells were treated with DMSO, 0.3 μM, or 5 μM NXP800 for 24 h. Data represent mean ± SEM of triplicate samples from two independent experiments and were normalized to cell number determined by Hoechst 33342 nuclear staining. Values were multiplied by 1×10^5 for visualization. Statistical significance compared with the DMSO control is indicated as *$p < 0.05$, **$p < 0.01$, ***$p < 0.001$, ****$p < 0.0001$ (two-way ANOVA).(A–B) Glycolytic Rate Assay profiles of HLF (A) and SNU449 (B) cells showing basal and compensatory glycolysis over time. Cells were sequentially treated with rotenone and antimycin A (Rot/AA) to inhibit mitochondrial respiration and reveal the glycolytic proton efflux rate (glycoPER), followed by 2-deoxy-D-glucose (2-DG) to inhibit glycolysis and confirm assay specificity. Left panels show glycoPER traces; right panels summarize basal glycolysis, compensatory glycolysis, basal proton efflux rate (PER), and post-2-DG acidification.(C–D) Mitochondrial Stress Test profiles of HLF (C) and SNU449 (D) cells showing oxygen consumption rate (OCR) over time. Cells were sequentially injected with oligomycin (ATP-linked respiration), carbonyl cyanide-4-phenylhydrazone (FCCP; maximal respiration and spare capacity), and Rot/AA (non-mitochondrial respiration). Left panels show OCR traces; right panels quantify basal respiration, maximal respiration, spare respiratory capacity, ATP production, and proton leak.

Uncropped blots of Figure 1

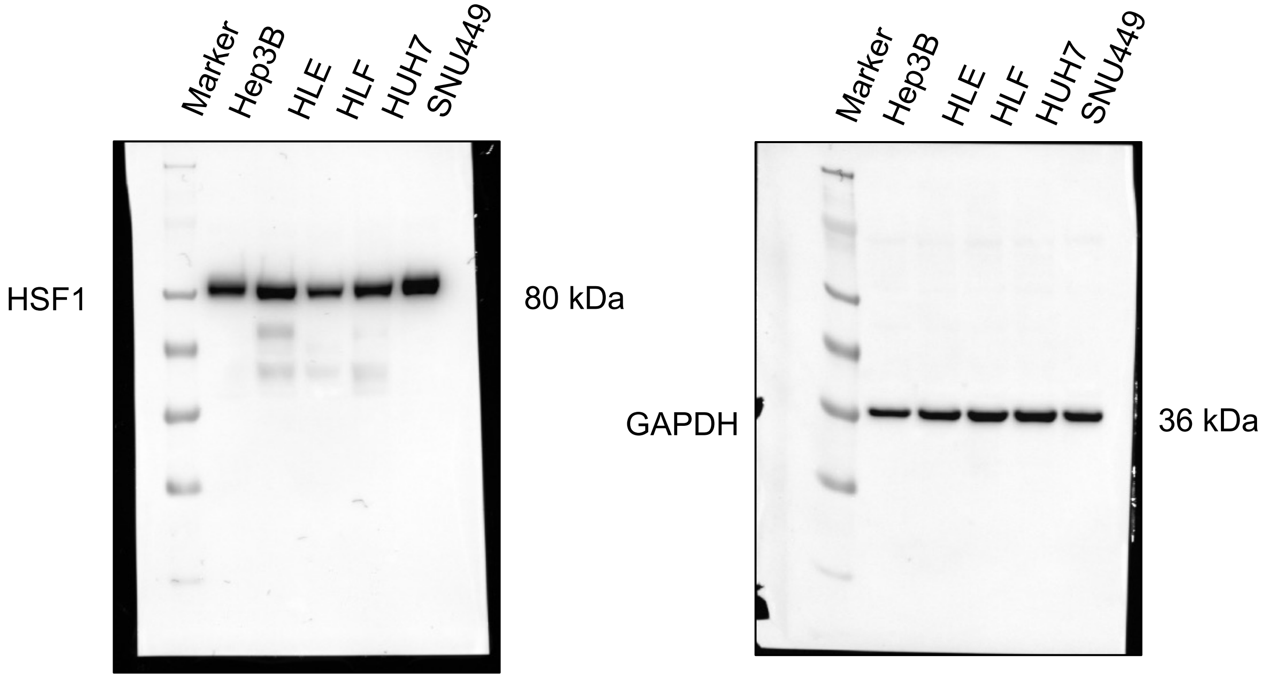

Uncropped blots of Supplementary Figure S2

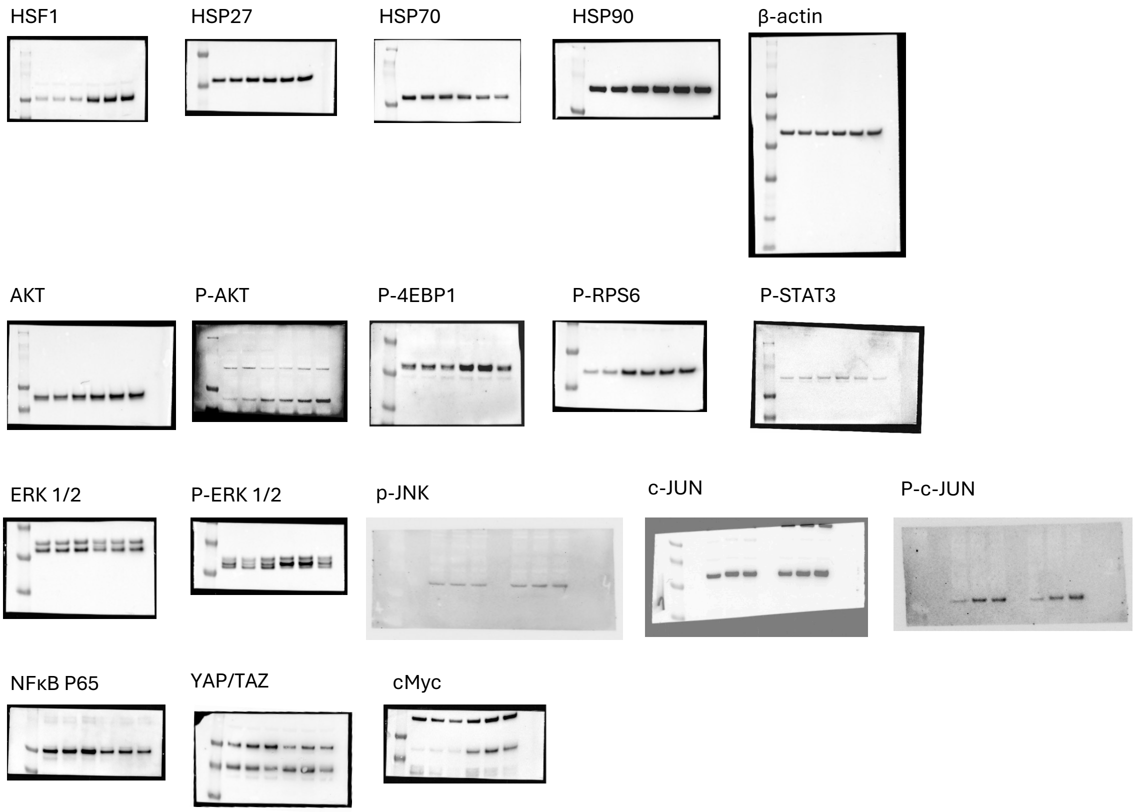

Supplementary Figure S4
